# Sequence-independent characterization of viruses based on the pattern of viral small RNAs produced by the host

**DOI:** 10.1093/nar/gkv587

**Published:** 2015-06-03

**Authors:** Eric Roberto Guimarães Rocha Aguiar, Roenick Proveti Olmo, Simona Paro, Flavia Viana Ferreira, Isaque João da Silva de Faria, Yaovi Mathias Honore Todjro, Francisco Pereira Lobo, Erna Geessien Kroon, Carine Meignin, Derek Gatherer, Jean-Luc Imler, João Trindade Marques

**Affiliations:** 1 Department of Biochemistry and Immunology, Instituto de Ciências Biológicas, Universidade Federal de Minas Gerais, Belo Horizonte, Minas Gerais, CEP 30270-901, Brazil; 2 CNRS-UPR9022, Institut de Biologie Moléculaire et Cellulaire, 67084 Strasbourg Cedex, France; 3 Department of Microbiology, Instituto de Ciências Biológicas, Universidade Federal de Minas Gerais, Belo Horizonte, Minas Gerais, CEP 30270-901, Brazil; 4 Laboratório Multiusuário de Bioinformática, Embrapa Informática Agropecuária, Campinas, São Paulo, CEP 13083-886, Brazil; 5 Faculté des Sciences de la Vie, Université de Strasbourg, 67083 Strasbourg Cedex, France; 6 Division of Biomedical and Life Sciences, Faculty of Health and Medicine, Lancaster University, Lancaster, Lancashire, LA1 4YQ, UK; 7 Institut d'Etudes Avancées de l'Université de Strasbourg (USIAS), 67084 Strasbourg Cedex, France

## Abstract

Virus surveillance in vector insects is potentially of great benefit to public health. Large-scale sequencing of small and long RNAs has previously been used to detect viruses, but without any formal comparison of different strategies. Furthermore, the identification of viral sequences largely depends on similarity searches against reference databases. Here, we developed a sequence-independent strategy based on virus-derived small RNAs produced by the host response, such as the RNA interference pathway. In insects, we compared sequences of small and long RNAs, demonstrating that viral sequences are enriched in the small RNA fraction. We also noted that the small RNA size profile is a unique signature for each virus and can be used to identify novel viral sequences without known relatives in reference databases. Using this strategy, we characterized six novel viruses in the viromes of laboratory fruit flies and wild populations of two insect vectors: mosquitoes and sandflies. We also show that the small RNA profile could be used to infer viral tropism for ovaries among other aspects of virus biology. Additionally, our results suggest that virus detection utilizing small RNAs can also be applied to vertebrates, although not as efficiently as to plants and insects.

## INTRODUCTION

Viruses are highly abundant in most biological systems and are characterized by an extraordinary diversity (1). Large-scale sequencing of RNA and DNA has been commonly used in metagenomic studies to assess the genetic diversity of viruses in a biological sample, referred to as the virome (1–5). In some cases, sample manipulation prior to sequencing, such as centrifugation and column filtration, are applied in order to enrich for viral sequences although such techniques can sometimes lead to contamination (6–8). Thus, direct nucleic acid extraction with few or no sample manipulation steps is the preferred strategy to minimize external contamination. However, the lack of viral enrichment may sometimes result in a majority of non-viral sequences in the library (9). Whether or not enrichment is employed, virus identification by metagenomics is inherently limited since it mostly relies on sequence similarity comparisons against reference databases. New strategies need to be developed to improve virus detection and help characterize novel unknown sequences commonly found in large-scale sequencing studies that are sometimes referred to as the ‘dark matter’ of metagenomics (10,11).

The characterization of insect viromes has particular public health significance since mosquitoes and other insect species can transmit human viral pathogens, such as *Dengue virus* and *Chikungunya virus* (12,13). Sequencing of small or long RNAs has been used to identify viruses in insects, although the potential advantages and disadvantages of each strategy are unclear (7,9,14–17). Notably, while long RNAs are direct products of viral replication and transcription, the biogenesis of small RNAs involves further processing of viral RNA products by host antiviral pathways such as RNA interference (RNAi). In insects and most animals, there are at least three different RNAi pathways that involve the production of distinct types of small RNAs, namely microRNAs (miRNAs), piwi-interacting RNAs (piRNAs) and small interfering RNAs (siRNAs). Each type of small RNA has a unique size distribution and nucleotide preference related to the RNAi pathway to which it belongs. In insects, RNA byproducts of viral replication can trigger the production of virus-derived small RNAs of length 21 and 24–29 nt, suggesting the activation of siRNA and piRNA pathways, respectively (15,18–21). Virus-derived siRNAs originate uniformly from both strands of genomes by processing of viral double-stranded RNA (dsRNA) generated in infected cells (18,22). The siRNA pathway is a major antiviral response against viruses containing DNA or RNA genomes since dsRNA seems to be a common by-product of viral replication (20,22–26). In contrast, virus-derived piRNAs that normally show a less uniform genome coverage can be generated from single-stranded RNA precursors but their function in controlling infection is less clear (20,21). Virus-derived siRNAs and piRNAs will associate with argonaute proteins to form the RNA-induced silencing complex that degrades complementary viral RNAs (18,22,23). In contrast to siRNAs and piRNAs, miRNAs are mostly derived from specific non-coding loci in the host genome and have no direct role in silencing of viral transcripts. Viruses can sometimes produce their own miRNAs but this seems to be mostly restricted to DNA viruses that infect vertebrate animals (27). In addition to the siRNA and piRNA pathways, other unrelated mechanisms can also generate virus-derived small RNAs from the degradation of viral RNAs, such as RNase L in mammals (28).

Here, we took advantage of the production of virus-derived small RNAs by the host response, to identify viruses within laboratory stocks of *Drosophila melanogaster* and wild populations of *Aedes aegypti* and *Lutzomyia longipalpis*. We show that small RNAs are relatively enriched and favour the detection of viral sequences compared to long RNAs in the same sample. This suggests that the production of virus-derived small RNAs by host antiviral pathways causes an enrichment of viral sequences in small RNAs compared to long RNAs. Moreover, we show that the size profile of small RNAs produced by host pathways is unique to each virus and can be used as a signature to classify and identify viral contigs independent of sequence similarity comparisons to known references. This pattern-based strategy overcomes a severe limitation of metagenomic approaches, allowing identification of novel viral contigs, which otherwise would have escaped detection by sequence-based methods. In addition, we noted that the small RNA profile could reflect aspects of virus biology since the activation of RNAi pathways is affected by viral genome structure and tissue tropism. We show that the profile of virus-derived small RNAs consistent with activation of the piRNA pathway in the germline, was successfully used to infer viral infection of mosquito ovaries. Using this small RNA based approach, we identified novel viruses from the *Bunyaviridae, Reoviridae* and *Nodaviridae* families that compose the virome of wild and laboratory insect populations. Using published small RNA datasets, we show that this strategy can also be broadly employed for the detection of viruses in animals and plants, although in vertebrates this application requires further validation.

## MATERIALS AND METHODS

### Sample processing and nucleic acid extraction

*Aedes aegypti* mosquitoes used on the experiments were obtained from laboratory colonies established from eggs collected in three neighbourhoods of Rio de Janeiro (Humaita, Tubiacanga and Belford Roxo), in southeastern Brazil. Laboratory colonies of *L. longipalpis* sandflies were derived from wild-caught animals captured in the city of Teresina, in northeastern Brazil. *Drosophila* libraries were prepared from wild-type laboratory stocks that were infected with *Vesicular stomatitis virus, Drosophila C virus* or *Sindbis virus* as described previously (29). Individual or pooled insects were anesthetized with carbon monoxide and directly ground in Trizol using glass beads. Ovaries were dissected from female mosquitoes and directly homogenized in Trizol reagent using a pipette. Total RNA or DNA was extracted using Trizol according to the manufacturer's protocol (Invitrogen).

### RNA library construction

Total RNA extracted from three separate pools of mosquitoes, sandflies and fruit flies were used to construct independent small RNA libraries. In the case of mosquitoes, the same total RNA was used to also construct three independent long RNA libraries. Small RNAs were selected by size (∼18–30 nt) on a denaturing PAGE before being used for construction of libraries as previously described (30). Long RNA libraries were constructed from total RNA that was poly(A) enriched and depleted for ribosomal RNA (rRNA) using a TruSeq Stranded Total RNA kit according to the manufacturer's protocol (Illumina). Sequencing was performed by the IGBMC Microarray and Sequencing platform, a member of the ‘France Génomique’ consortium (ANR-10-INBS-0009). Sequence strategy was 1 × 50 base pairs (bp) for small RNAs libraries and 2 × 100 bp (forward and reverse sequencing) resulting in an average read length of 190 nt total.

### Pre-processing of RNA libraries

Raw sequenced reads from small RNA libraries were submitted to quality filtering and adaptor trimming using fastx_quality_filter and fastq_clipper respectively, both part of the fastx-toolkit package (version 0.0.14) (http://hannonlab.cshl.edu/fastx_toolkit/index.html). Small RNA reads with phred quality below 20, shorter than 15 nt after trimming of adaptors or containing ambiguous bases, were discarded. In the case of long RNA libraries, raw sequenced reads were submitted to quality filtering using fastx_quality_filter. Reads with phred quality below 20 or containing ambiguous bases were discarded. Remaining sequences from small or long RNA libraries were mapped to reference sequences from transposable elements, bacterial genomes (2739 complete genomes deposited in Genbank) and host genomes (*L. longipalpis, A. aegypti* and *D. melanogaster*) using Bowtie (version 1.1.1) for small RNA libraries or Bowtie2 (version 2.2.4) for long RNA libraries (one mismatch allowed) (31,32). The *Drosophila* genome (version v5.44) was downloaded from flybase.org. The latest versions of mosquito (Liverpool strain L3) and sandfly (Jacobina strain J1) genomes were downloaded from VectorBase (https://www.vectorbase.org/). Sequences of transposable elements were obtained from TEfam (http://tefam.biochem.vt.edu/tefam/). Remaining sequenced reads that did not map to transposable elements, host or bacterial genomes, referred to as processed reads, were used for contig assembly and subsequent analysis.

### Contig assembly strategy

Processed reads were utilized for contig assembly using Velvet (version 1.0.13) (33). Assembly was performed in parallel using different strategies for each library. For small RNA libraries, we performed contig assembly using different size ranges of small RNA reads (20–23, 24–30 and 20–30 nt). In each case, parallel assembly strategies were performed using a fixed k-mer value (k-mer 15) with default parameters or a k-mer value between 15 and 31 defined automatically by VelvetOptimser (version 2.2.5) (http://bioinformatics.net.au/software.velvetoptimiser.shtml). For long RNA libraries, contig assembly was performed using a fixed k-mer value (k-mer 31) or an automatically defined k-mer value from 15 to 91. For each library, results from parallel contig assembly strategies were merged using CAP3 (version date 12-21-07) with max gap length in overlap of 2 and overlap length cutoff of 20 (34). Results from assembly strategies utilizing different size ranges of small RNAs were also combined using CAP3. Removal of redundant contig sequences was performed using BLASTClust program within the standalone BLAST package (version 4.0d) (35), requiring 50% of length with at least 50% of identity between contigs. Non-redundant contigs larger than 50 nt received specific IDs to indicate their origin and were further characterized.

### Sequence-based characterization of contigs

Assembled contigs were characterized by sequence similarity (nucleotide and protein) to known sequences, with analysis of conserved domains if detected, and also examined for the presence of ORFs. We used BLAST for sequence similarity searches against non-redundant NCBI databases (nucleotides and protein). InterproScan (version 5.3–46.0) (36) and HMMer (version 3.0) (37) were used to verify the presence of open reading frames (ORFs) and conserved domains and the Pfam database (version 27.0) (38) to analyse protein domains. Hits with an *E*-value smaller than 1e^−5^ for nucleotide comparison or 1e^−3^ for protein comparison were considered significant. Viral genomic segments were classified as described (39).

### Analysis of small RNA profiles

For pattern-based analysis, processed small RNA reads were mapped against contig or reference sequences using Bowtie allowing one mismatch. Small RNA size profile was calculated as the frequency of each small RNA read size from 15–35 nt mapped on the reference genome or contig sequence considering each polarity separately. We used a Z-score to normalize the small RNA size profile and to plot heatmaps for each contig or reference sequence using R (version 3.0.3) with gplots package (version 2.16.0). To evaluate the relationship between small RNA profiles from different contig or reference sequences, we computed the Pearson correlation (confidence interval >95%) of the Z-score values. Similarities between small RNA profiles were defined using hierarchical clustering with UPGMA as the linkage criterion. Groups of sequences with more than one element with at least 0.8 of Pearson correlation between each other were assigned to clusters. The density of small RNA coverage was calculated as the number of times that small RNA reads covered each nucleotide on the reference genome or contig sequence. Small RNA size profile and density of coverage were calculated using in-house Perl (version 5.12.4) scripts using BioPerl (version 1.6.923) and plotted using R with ggplot2 (version 1.0.1).

### RT-PCR and Sanger sequencing

About 200 ng of total RNA extracted from insects was reverse transcribed into cDNA using MMLV reverse transcriptase. cDNA or DNA were subjected to polymerase chain reaction (PCR) reactions using specific primers. Oligonucleotide primers are listed in Supplementary Table S1 and were designed according to contig sequences obtained from our assembly pipeline. PCR products were subjected to direct Sanger sequencing.

## RESULTS

### Optimizing contig assembly from small RNA sequences

**Figure 1.**
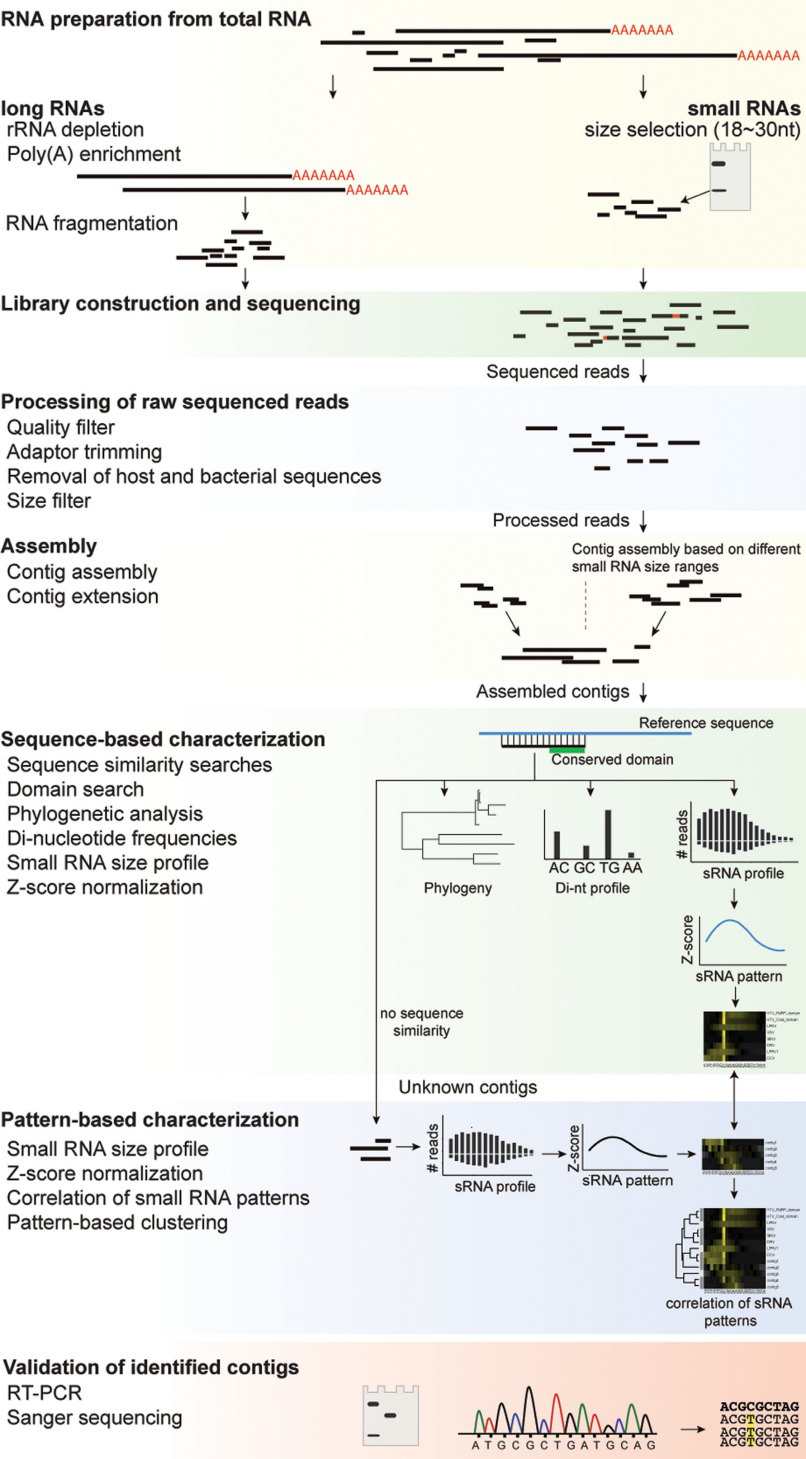
Overview of the pipeline for virus detection based on long and small RNAs. Different RNA fractions were utilized for the construction of small and long RNA libraries. Sequenced reads were processed to enrich for potential virus sequences. Processed reads were then utilized for contig assembly and extension. Contigs were characterized using both sequence-based and pattern-based strategies. Viral contigs were further validated by RT-PCR and Sanger sequencing. See text for details.

**Figure 2.**
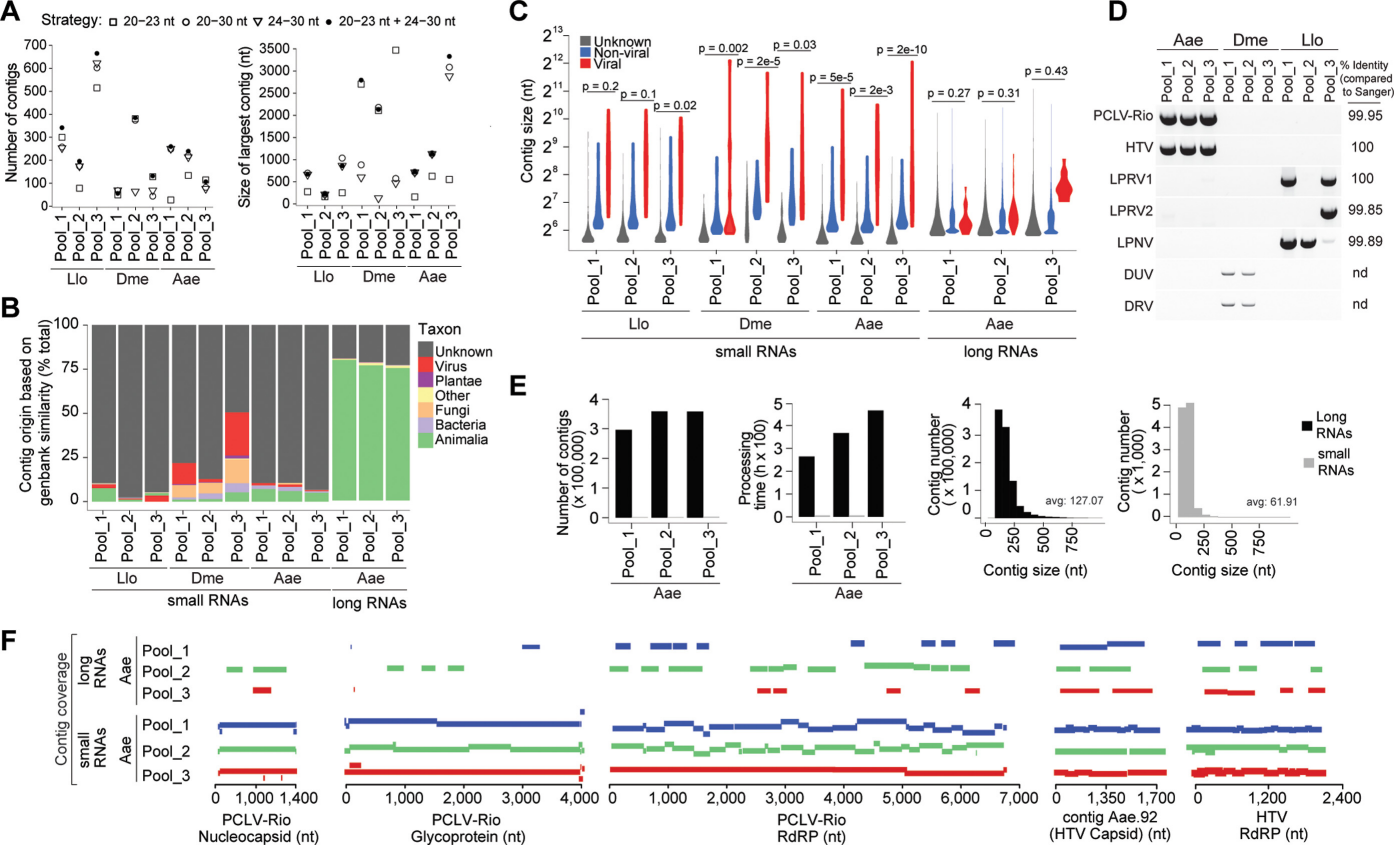
Small RNA sequencing identifies viral sequences more efficiently than long RNAs. (**A**) Comparison of number of contigs and size of largest contig in each small RNA library using different size ranges of small RNAs in the assembly step. (**B**) Proportion of contigs assembled in each library with significant similarity to reference sequences. The origin of contigs is classified by taxon and includes unknown sequences. (**C**) Size distribution of viral (red), non-viral (blue) and unknown contigs (grey) for each library. *P*-values for the difference between viral and non-viral contig sizes are indicated (Student *t*-test). (**D**) Viral RNA sequences were detected by RT-PCR from total RNA extracted from three separate pools of *Drosophila, Aedes* and *Lutzomyia* populations. Sanger sequencing of PCR products showed high identity to the sequence determined by our metagenomics approach as shown in the right column (not done; nd). (**E**) Comparison of processing time, number of contigs and frequency distribution of contig sizes for small and long RNA libraries shown in grey and black, respectively. (**F**) Coverage of PCLV and HTV genome segments by contigs assembled in each small and long RNA libraries from mosquitoes. Biological replicate samples are shown in blue, green and red.

Large scale sequencing of small RNAs has been used for virus identification in insects and plants (15,16). Thus, we constructed small RNA libraries from laboratory stocks of *D. melanogaster* and wild populations of *A. aegypti* mosquitoes and *L. longipalpis* sandflies, two important vectors for human pathogens (Supplementary Table S2). *Drosophila* libraries were prepared from laboratory strains infected with three distinct viruses, *Drosophila C virus* (DCV), *Sindbis virus* (SINV) and *Vesicular stomatitis virus* (VSV) in order to help optimize our virus detection pipeline for small RNA sequences. Small RNA libraries were prepared from whole insects with no sample manipulation prior to RNA extraction to minimize risks of sample contamination in the laboratory, which is essential when processing field samples. After sequencing of small RNA libraries, data were processed to enrich for potential viral sequences by removing sequences derived from host and bacterial genomes. Host sequences corresponded to the vast majority of small RNAs (73–92%) in our libraries but a substantial percentage of sequences (3.4–14% of libraries) remained after these processing steps (Figure 1). However, these are short sequences that need to be assembled into longer contiguous sequences (contigs) before being used for sequence-similarity searches against reference databases. Several studies have shown that virus-derived small RNAs are mostly 21 nt-long siRNAs (18,22,23). However, we reasoned that focusing on 21-nt long small RNAs could be shortsighted. Indeed, the piRNA pathway or degradation by other exonucleases may also generate virus-derived small RNAs in insect hosts (15,20,21). Importantly, these small RNAs of different origins could cooperate to allow the assembly of longer contigs. Therefore, we tested the use of different size ranges of small RNAs for contig assembly (Figure 2A). The best number and largest size of contigs were obtained when 20–23 and 24–30 nt small RNAs were utilized to assemble contigs separately and results combined afterwards (Figure 2A). Contig assembly utilizing other small RNA size ranges including 20–23, 24–30 or 20–30 nt small RNAs resulted in variable metrics depending on the library. Thus, the combination of contig assembly results utilizing 20–23 and 24–30 nt small RNAs separately seems to be more broadly applicable without prior knowledge of the small RNA profile.

Libraries from infected *Drosophila* were used to directly assess our virus detection strategy. In these libraries, we detected 42, 40 and 1 contigs that showed significant similarity against VSV, SINV and DCV, respectively (Supplementary Figure S1A). Thus, our approach could detect viruses known to be present in flies, although detection was limited by the number of viral small RNAs. We observed 1572 small RNAs derived from DCV that only allowed assembly of one contig covering 0.8% of the viral genome (Supplementary Figure S1). In contrast, 53 620 and 9588 small RNAs derived from VSV and SINV, respectively, allowed assembly of multiple independent contigs that covered 81.1 and 23.4% of the respective genomes (Supplementary Figure S1A). Thus, high coverage of viral genomes is important to allow contig assembly from overlapping small RNAs.

Next, all unique contigs assembled from *D. melanogaster, A. aegypti* and *L. longipalpis* small RNA libraries were utilized for sequence similarity searches against the NCBI non-redundant databases (nucleotide and protein). The vast majority of contigs assembled in all nine small RNA libraries (10 577 out of 11 806) did not show any significant sequence similarity and are hereafter referred to as unknown (Figure 2B). The large majority of these contigs (92%) are shorter than 100 nt thus hampering more detailed analyses. Nevertheless, clustering of our libraries based on the similarity of unknown sequences separates *Drosophila, Aedes* and *Lutzomyia* samples, suggesting that these contigs are host-specific (Supplementary Figure S2). The remaining 1229 non-redundant contigs were classified according to the taxon assigned to their most significant BLAST hit (Figure 2B). Several contigs showed similarity to animal sequences especially in the case of mosquito and sandfly libraries (Figure 2B). These likely belong to the insect genome but were not successfully removed in the pre-processing step. This may reflect the fact that the genomes of *A. aegypti* and *L. longipalpis* are not as well curated as the *D. melanogaster* genome (40,41). Several of the remaining contigs are derived from bacteria and fungi and could be part of the insect microbiome.

### Sequence-based detection of viruses in contigs assembled from small RNAs

Out of 1229 non-redundant contigs, 223 (∼18%) showed significant similarity to viral sequences in reference databases (Figure 2B and Supplementary Table S2). The mean size of viral contigs was significantly longer than all the rest and included all the largest assembled sequences (Figure 2C). These results suggest that our small RNA based strategy favours assembly of long viral contigs compared to sequences of other origin. We removed 83 contigs derived from DCV, SINV or VSV that were among the 223 viral contigs. The remaining 140 viral contigs were filtered to eliminate similar sequences detected in more than one library from the same insect species. We also used overlap between contigs to further extend viral sequences. These steps allowed us to generate merged results from the three independent small RNA libraries from each insect population, *Drosophila, Aedes* and *Lutzomyia*. We were able to reduce 140 total viral contigs to 34 non-redundant sequences that could be assigned to at least seven viruses based on the most significant BLAST hit in reference databases (Table 1). Phylogenetic analysis suggests that six out of the seven viruses represent completely new species. Regarding the virome of each insect species, two viruses were detected in mosquitoes, three in sandflies and two in fruit flies.

**Table 1.** Summary of viruses identified in *Drosophila melanogaster, Aedes aegypti* and *Lutzomyia longipalpis*

Host	Virus family	Virus	Largest contig (nt)	Segment status^1^	# contig (sum of libraries)	ID strategy	Best hit	*E*-value	Accession number (size of reference in nt)
*A. aegypti*	*Bunyaviridae*	PCLV	3936	CC	4	blastx	glycoprotein precursor [Phasi Charoen-like virus]	0E + 00	AIF71031.1 (3852)
		PCLV	6807	CC	23	blastx	RdRP [Phasi Charoen-like virus]	0E + 00	AIF71030.1 (6783)
		PCLV	1332	CC	3	blastx	nucleocapsid [Phasi Charoen-like virus]	2E − 72	AIF71032.1 (1398)
	*Unassigned*	HTV	1609	CC	8	blastx	structural protein precursor [Drosophila A virus]	2E − 65	YP_003038596.1 (1326)
		HTV	2793	CC	13	blastx	putative RdRP [Laem Singh virus]	8E − 34	AAZ95951.1 (507)
*L. longipalpis*	*Reoviridae*	LPRV1	3762	CC	11	blastx	RdRP [Choristoneura occidentalis cypovirus 16]	3E − 173	ACA53380.1 (3675)
		LPRV1	3687	CC	5	blastx	VP3 [Inachis io cypovirus 2]	1E − 81	YP_009002593.1 (3450)
		LPRV1	3200	CC	2	blastx	VP4 [Inachis io cypovirus 2]	4E − 63	YP_009002588.1 (3201)
		LPRV1	1842	CC	2	blastx	VP5 [Inachis io cypovirus 2]	2E − 16	YP_009002589.1 (1899)
		LPRV1	841	CC	1	blastx	polyhedrin [Simulium ubiquitum cypovirus]	6E − 69	ABH85367.1 (836)
		LPRV1	3685	CC	1	blastx	VP2 [Inachis io cypovirus 2]	5E − 24	YP_009002587.1 (3649)
		LPRV1	1547	HQ	2	phmmer	unknown [Choristoneura occidentalis cypovirus 16]	900E − 03	ABW87641.1 (1946)
		LPRV1	2237	CC	3	blastx	unknown [Choristoneura occidentalis cypovirus 16]	200E − 01	ABW87640.1 (2214)
		LPRV1	2231	CC	1	pattern-based			
		LPRV1	1345	CC	1	pattern-based			
		LPRV1	688	HQ	1	pattern-based			
		LPRV1	680	HQ	1	pattern-based			
		LPRV2	3680	CC	1	blastx	RdRP [Bombyx mori cypovirus 1]	0E+00	AAK20302.1 (3854)
		LPRV2	1116	CC	1	blastx	polyhedrin [Heliothis armigera cypovirus 14]	4E-11	AAY34355.1 (956)
		LPRV2	2043 + 779 + 1392	SD	3	blastx	VP1 protein [Dendrolimus punctatus cypovirus 1]	4E-70	AAN84544.1 (4164)
		LPRV2	964	HQ	1	blastx	hypothetical protein LdcV14s9gp1 [Cypovirus 14]	2E-09	NP_149143.1 (1141)
		LPRV2	678 +1035 + 1617	SD	3	blastx	VP3 [Bombyx mori cypovirus 1]	5E-14	ADB95943.1 (3262)
		LPRV2	443 + 579 + 769	SD	3	blastx	viral structural protein 4 [Bombyx mori cypovirus 1]	2E-10	ACT78457.1 (1796)
		LPRV2	1516	HQ	1	blastx	VP2 protein [Dendrolimus punctatus cypovirus 1]	8E-53	AAN86620.1 (3846)
		LPRV2	599	HQ	4	blastx	unknown [Operophtera brumata cypovirus 18]	4E-10	ABB17215.1 (2883)
		LPRV2	286	HQ	2	blastx	putative VP5 [Dendrolimus punctatus cypovirus 1]	3E-02	AAO61786.1 (1501)
		LPRV2	641	SD	1	pattern-based			
		LPRV2	1212	SD	1	pattern-based			
		LPRV2	1174	CC	1	pattern-based			
		LPRV2	976	SD	1	pattern-based			
		LPRV2	535	SD	1	pattern-based			
	*Nodaviridae*	LPNV	2054	CC	5	blastx	capsid protein [Nudaurelia capensis beta virus]	1E − 42	NP_048060.1 (1836)
		LPNV	3189	CC	23	blastx	RdRP [Nodamura virus]	9E − 82	NP_077730.1 (3129)
*D. melanogaster*	*Unassigned*	DUV	1905 + 452	SD	2	blastx	protein P1 (RdRP) [Acyrthosiphon pisum virus]	2E −63	NP_620557.1 (10 035)
	*Reoviridae*	DRV	635 + 175	SD	2	blastx	RdRP [Fiji disease virus]	8E − 05	YP_249762.1 (4532)

^1^Segment status defined as described by Ladner *et al*. (39): SD: Standard Draft, HQ: High quality, CC: Coding complete, C: Complete, F: Finished.

In *Aedes* mosquitoes we detected contigs that belong to a novel strain of *Phasi Charoen Like-virus* (PCLV), a bunyavirus previously identified in mosquitoes from Thailand (Supplementary Figure S3A)(42). In addition, we also identified contigs from a novel virus related to *Laem Singh virus* (LSV) and two other recently described tick viruses, *Ixodes scapularis associated virus* 1 and 2 (Supplementary Figure S3B) (7), all of which are taxonomically unclassified. This new virus was named *Humaita-Tubiacanga virus* (HTV) to reflect the origin of its host.

In sandflies, we observed several non-redundant contigs showing similarity to reoviruses and nodaviruses (Table 1). Specifically, 23 non-redundant contigs showed sequence similarity to viruses of the genus *Cypovirus* from the family *Reoviridae* (Table 1). This number of unique viral contigs is high even considering the fact that reoviruses can have up to 12 genomic segments. Based on the phylogenetic analysis of genomic segments encoding viral RNA-dependent RNA polymerases (RdRPs), we were able to identify two distinct reoviral sequences that belong to the genus *Cypovirus* (Supplementary Figure S3C). These viruses were named *Lutzomyia Piaui reovirus* 1 (LPRV1) and *Lutzomyia Piaui reovirus 2* (LPRV2) to reflect their host and geographical location. Analyses of the other viral contigs assembled from sandfly libraries that showed similarity to nodaviruses suggest they belong to a novel virus related to *Nodamura virus* and a member of the genus *Alphanodavirus* (Supplementary Figure S3D). This novel virus was named *Lutzomyia Piaui nodavirus* (LPNV).

In fruit flies, we detected contigs that showed similarity to two viral families unrelated to the viruses used for experimental infections. That suggested the *Drosophila* laboratory stocks we used already carried unrecognized viral infections (Table 1). One set of contigs showed similarity to reoviruses. Phylogeny suggests these belong to a virus of the genus *Fijivirus* of the family *Reoviridae* (Supplementary Figure S3C). This virus was named *Drosophila reovirus* (DRV). Another viral contig showed similarity to *Acyrthosiphon pisum virus* but could not be assigned to any known viral families by phylogeny (Supplementary Figure S3E). This virus was consequently named *Drosophila uncharacterized virus* (DUV).

Sequences corresponding to all seven potential new viruses were successfully amplified by PCR from reverse transcribed RNA but not from DNA (Figure 2D and data not shown). This indicates they are present in an RNA form, which is consistent with the observation that they presumably belong to viral families with RNA genomes (Figure 2D and Table 1). Sanger sequencing of PCR products showed 99–100% sequence identity to the contigs assembled using our strategy (Figure 2D). Importantly, all single nucleotide differences were also present in small RNA sequences from the individual libraries prior to contig assembly suggesting natural variations in virus populations (data not shown). Notably, the presence of these seven viruses was only detected in the corresponding insect populations utilized for the construction of small RNA libraries where they were first identified, *Drosophila, Aedes* or *Lutzomyia*. These results indicate that our strategy is not prone to generate artifacts.

### Small RNAs are naturally enriched for viral sequences compared to long RNAs

We successfully detected seven viruses using small RNA libraries but had no basis to compare how this strategy would fare against other alternatives for detection of viral sequences. Other strategies utilize some type of sample manipulation in order to enrich for viral sequences prior to nucleic acid extraction although this may result in contamination (7,9). Direct sequencing of long RNAs is also utilized but can be limited by the abundance of host rRNA molecules that represent the vast majority of sequences in long RNA libraries (43). As an alternative, we constructed long RNA libraries after rRNA depletion and poly(A) enrichment from the same total RNA of *A. aegypti* populations used to prepare small RNA libraries (Supplementary Table S2). This allowed us to directly compare results from large scale sequencing of small and long RNAs from the same samples without manipulation prior to RNA extraction. The number and length of sequences obtained with long RNA libraries resulted in 10.4-fold more data compared to small RNA libraries. As a result, long RNA libraries generated a total of 1 011 347 contigs with N50 of ∼136 nt compared to 6066 contigs with N50 of ∼48 nt for small RNA libraries (Figure 2E and Supplementary Table S2). The larger number of contigs resulted in 43- to 72-fold longer processing times for similarity searches against databases comparing long and small RNA libraries (Figure 2E). Most contigs assembled from long RNA libraries (>60%) showed similarity to animal sequences and are likely to be unassembled parts of the *A. aegypti* genome (Figure 2B). Long RNA libraries also contained a large number of unknown sequences but they did not represent the majority of contigs as observed for small RNA libraries (Figure 2B). These results would suggest that long RNA libraries are more indicated for virus detection since they had significantly more contigs. However, the total number of viral contigs was very similar in small and long RNA libraries (Supplementary Table S2). Furthermore, the average size of viral contigs was longer in small RNA libraries, which resulted in larger coverage of viral genomes in all three independent samples (Figure 2C and F). Even though both strategies allowed detection of the same viruses, PCLV and HTV, these results indicate that small RNA libraries were enriched and naturally favoured assembly of viral sequences compared to long RNAs. The mechanism of small RNA biogenesis by host pathways appears to favour the generation of overlapping sequences that are likely to be important in allowing significant contig extension compared to sequencing of long RNAs. It is possible that viral RNAs could be further enriched in long RNAs had we not limited our sequencing to polyadenylated RNA molecules. Nevertheless, rRNA depletion alone could still bias sequencing results and small RNAs show natural enrichment for viral sequences without extensive processing steps prior to library construction.

### Classifying viral sequences using small RNA pattern analysis

**Figure 3.**
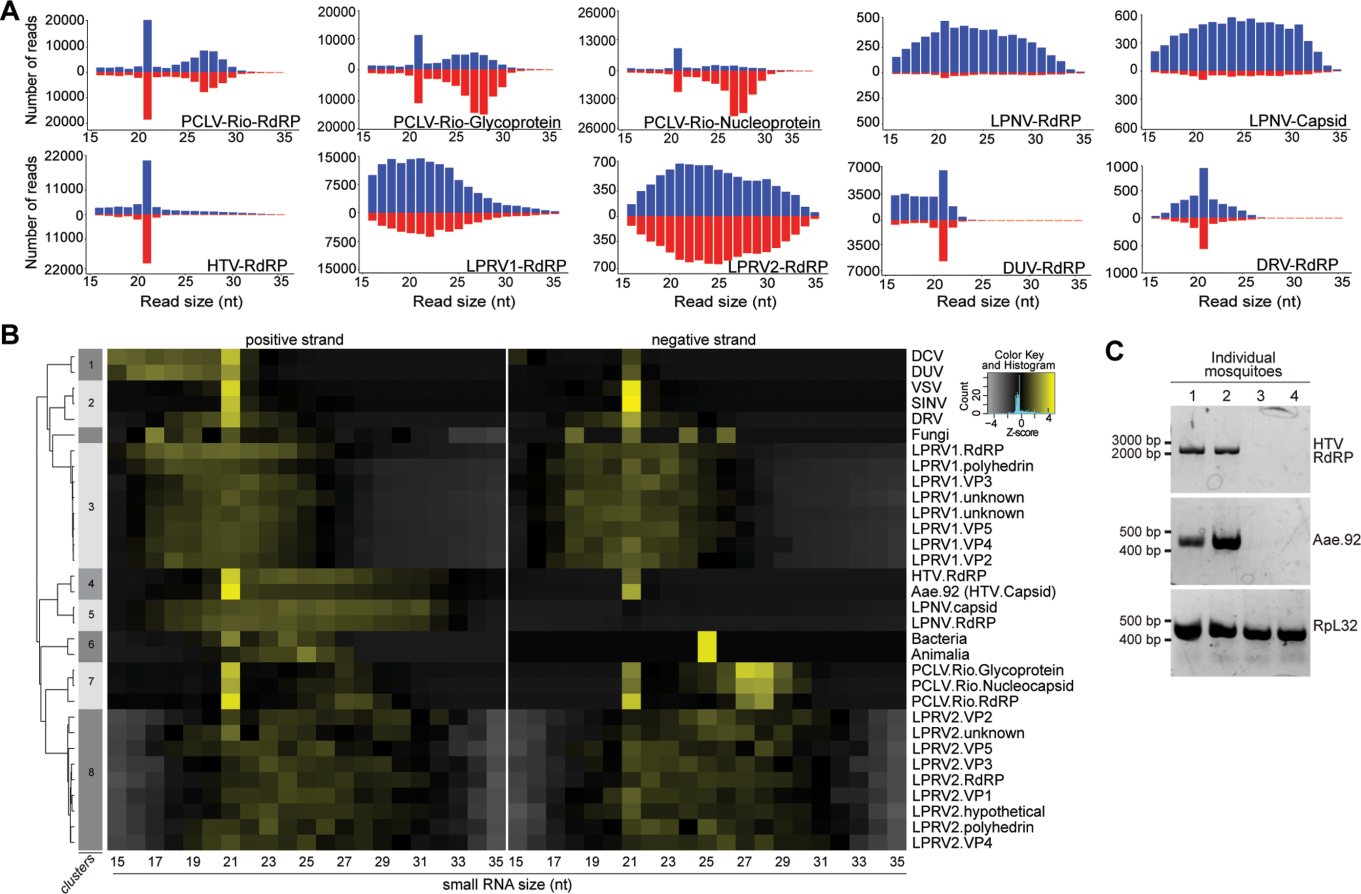
Small RNA size profile can classify uncharacterized viral contigs. (**A**) Small RNA size profile of previously characterized virus segments identified by sequence similarity searches. Blue and red represent small RNAs in the positive and negative strands, respectively. (**B**) Hierarchical clustering of viral contig sequences assembled in fruit fly, mosquito and sandfly libraries. Clustering was based on Pearson correlation of small RNA size profile shown as a heatmap. Clusters with more than one contig are indicated on the left vertical bar and numbered according to the order in which they appear from top to bottom. Clusters were defined by Pearson correlation above 0.8. (**C**) Contig Aae.92 and the segment corresponding to the HTV RdRP that grouped together by similarity of the small RNA size profile in panel (B) show perfect correlation of expression in individual mosquitoes as determined by RT-PCR. Results are representative of 46 individual mosquitoes that were analysed. The endogenous gene *Rpl32* was used as control for the RT-PCR.

Our results indicate that small RNAs libraries favour the detection of viruses compared to long RNAs. However, the majority of contigs assembled from small RNAs were not identified by sequence similarity searches against reference databases. Sequence independent strategies are necessary to identify highly divergent viruses that have no known relatives. The size profile of virus derived small RNAs produced by the host pathways was unique for each virus analysed in this study including PCLV, SINV, VSV, DCV, HTV, LPNV, LPRV1, LPRV2, DRV and DUV (Figure 3A and Supplementary Figure S1B). Additionally, small RNA size profiles observed for contigs derived from other organisms such as Fungi and Bacteria were also very distinct (Supplementary Figure S4). In the case of segmented viral genomes, the small RNA size profile was remarkably similar for different segments of the same virus such as PCLV and LPNV (Figure 3A). These small RNA size profiles were consistent with diverse origins of small RNAs including production of siRNAs (peaks at 21 nt), piRNAs (peak from 27–28 nt) or degradation of viral RNAs (no size enrichment, strong bias for small RNAs corresponding to the genomic strand) but are hard to classify visually (Figure 3A and Supplementary Figure S1B). Thus, we used a Z-score to normalize the small RNA size profile and generate heatmaps for each contig that could be subjected to hierarchical clustering based on pairwise correlations to evaluate their relationship (Figure 3A). Using this strategy, small RNA size profiles of different viruses usually showed low correlation. By contrast, the small, medium and large segments of PCLV were grouped in a common cluster of similarity (cluster 7) as well as the RdRP and capsid segments of LPNV (cluster 5) (Figure 3B). Since contigs representing genomic segments of the same virus were grouped together, we tested whether the correlation of small RNA size profiles could help classify additional contigs that showed similarity to viral sequences but could not be further characterized.

In sandflies, based on the analysis of sequences encoding viral RdRPs, we were able to identify two separate reoviruses, namely LPRV1 and LPRV2. However, we observed another 21 non-redundant contigs showing similarity to reoviruses that could not be assigned to LPRV1 or LPRV2 solely based on sequence similarity. Since the small RNA size profile of LPRV1 and LPRV2 RdRP segments were clearly distinct (Figure 3A), we hypothesized it could be used to classify the origin of the remaining 21 reovirus contigs. Using this strategy, we observed that seven reovirus contigs were grouped together with the RdRP of LPRV1 (cluster 3) while 8 formed a cluster with LPRV2 RdRP (cluster 8) based on the similarity of the small RNA size profile (Table 1 and Figure 3B). Thus we analysed the expression of contigs in clusters 3 and 8 compared to the RdRPs of LPRV1 and LPRV2. Consistent with the small RNA profile similarity, contigs in cluster 3 are detected in the same libraries as the LPRV1 RdRP while contigs in cluster 8 follow the expression of the RdRP of LPRV2 (data not shown).

In mosquitoes, we identified one viral contig (Aae.92) of 1609 nt predicted to encode a protein with a coat domain (PF00729). This contig showed similarity with the capsid protein of *Drosophila A virus* (DAV) but phylogenetic analysis suggests the two viruses are considerably distinct (Supplementary Figure S5A). Phylogenetic analysis would seem to suggest that contig Aae.92 belongs to a viral family distinct from the two viruses that were also found in mosquitoes, PCLV and HTV. However, we note that DAV is an unusual virus, whose RdRP and capsid proteins show similarity to different viral families (Supplementary Figure S5) (44). Notably, the small RNA size profile for the HTV RdRP and contig Aae.92 were remarkably similar and clustered together based on correlation of the small RNA size profile (Figure 3B). Hence, we hypothesized that contig Aae.92 encodes the capsid protein of HTV as we only characterized a segment corresponding to the RdRP of this virus. In agreement with this hypothesis, we observed 100% correlation between the detection by RT-PCR of contig Aae.92 and the RdRP segment of HTV in individual mosquitoes (Figure 3C and data not shown).

### A pattern-based strategy that identifies viral contigs in a sequence-independent manner

**Figure 4.**
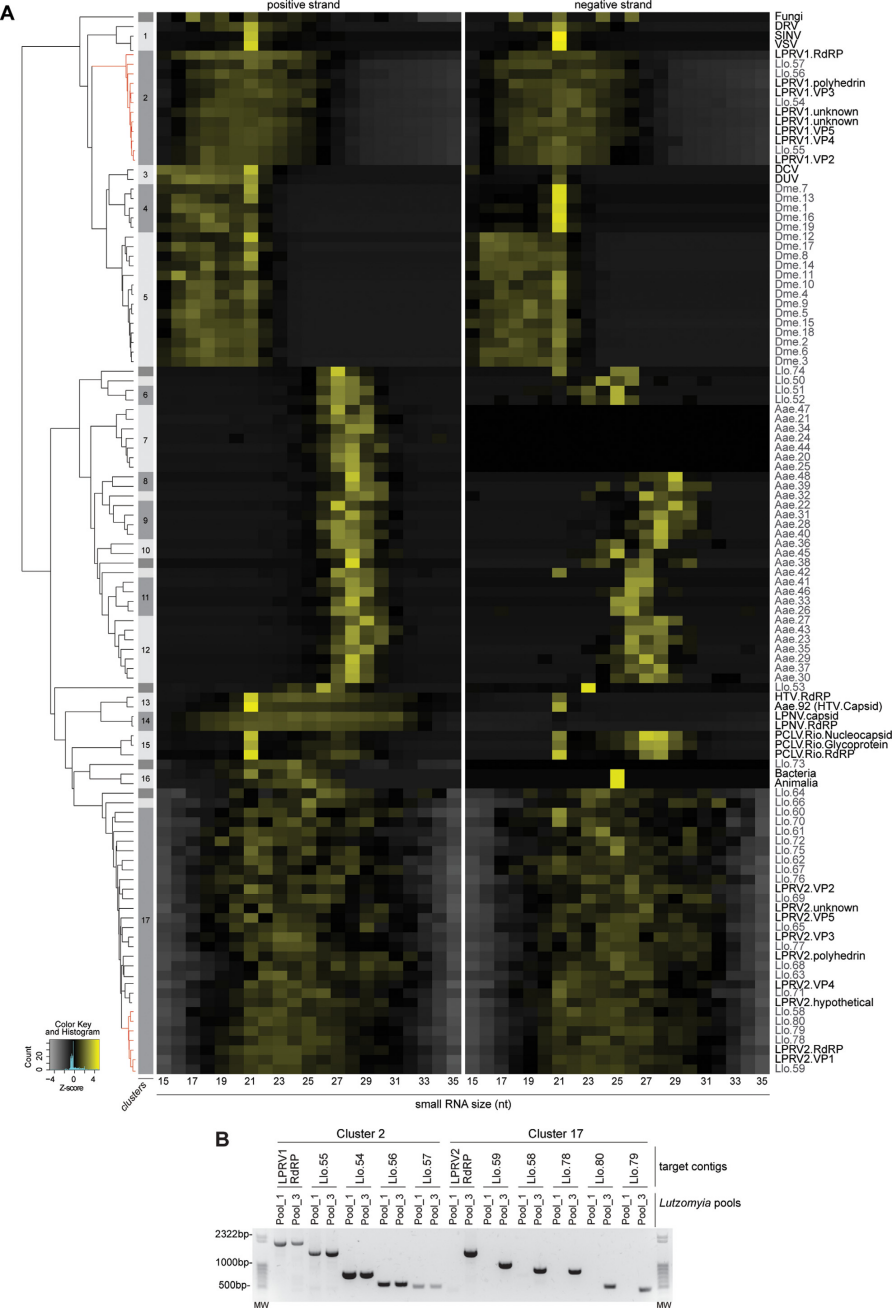
Small RNA pattern-based analysis identifies viral contigs without known relatives in reference databases. (**A**) Hierarchical clustering of viral and unknown contig sequences assembled in fruit fly, mosquito and sandfly libraries. Clustering was based on Pearson correlation of the small RNA size profile shown as a heatmap. Clusters with more than one contig are indicated on the left vertical bar and numbered according to the order in which they appear from top to bottom. Clusters were defined by Pearson correlation above 0.8. (**B**) Detection by RT-PCR in two separate pools of sandflies shows that contig sequences in Clusters 2 and 17 mimic the expression of RdRP segments of LPRV1 or LPRV2, respectively. The same pools of *Lutzomyia longipalpis* (pool1 and pool3) analysed in Figure 2D were used.

Viral contigs show unique small RNA size profiles that can be used to assign sequences to specific viruses in our samples. Possibly, this pattern analysis strategy could also help identify unknown contigs independently of sequence-similarity searches. In order to select prospective unknown contigs to be analysed, we noted that viral contigs were the largest assembled in our small RNA libraries (N50 of 208 nt compared to 63 nt for non-viral contigs) (Figure 2C). Thus, we used N50 as a proxy to filter 10 577 contigs representing unknown sequences and select 106 candidates longer than 208 nt. We eliminated sequence redundancy among these candidates, which resulted in 79 unique unknown contigs that were labelled according to their library of origin, *Lutzomyia* (Llo), *Drosophila* (Dme) or *Aedes* (Aae). Small RNA size profile was determined for all 79 unknown contigs and compared to previously characterize viral contigs using hierarchical clustering. This analysis generated 17 clusters containing more than one element, which were numbered sequentially according to the position in the heatmap. We observed that 72 out of the 79 unique unknown contigs were grouped in 11 different clusters of similarity (Figure 4A). Interestingly, clusters were composed of contigs assembled exclusively in libraries from the same insect, *Lutzomyia, Drosophila* or *Aedes*.

Unknown contigs found in *Lutzomyia* libraries were grouped in three separate clusters that showed clearly distinct small RNA patterns (Clusters 2, 6 and 17 in Figure 4A). Cluster 6 contained contigs with small RNA size profiles consistent with insect piRNAs (peak size between 27–28 nt) suggesting they could be derived from transposable elements (Figure 4A). Cluster 2 contained four unknown contigs that were grouped together and showed high correlation to previously identified LPRV1 segments (highlighted in red). Cluster 17 contained 19 unknown contigs that showed good correlation to LPRV2 segments (Figure 4A). Notably, in cluster 17, we observed that 5 of the 19 contigs formed a subgroup with correlation higher than 0.93 to LPRV2 RdRP segment (highlighted in red). These results suggest that some of the unknown contigs in *Lutzomyia* libraries could actually represent additional segments of LPRV1 and LRPV2. Indeed, based on the multi-segmented nature of reovirus genomes, we expected to find more segments for both LPRVs than were detected by sequence similarity searches (Table 1). In order to investigate this possibility, we analysed the expression of selected unknown contigs highlighted in cluster 2 and cluster 17 that presented the highest correlation to the small RNA size profile of RdRP segments from LPRV1 or LPRV2, respectively. All four unknown contigs in cluster 2 perfectly mimicked the expression profile of the RdRP segment from LPRV1 while all five contigs from cluster 17 copied the expression of LPRV2 (Figure 4B). None of these nine new LPRV contigs showed significant similarity to reovirus sequences in reference databases, suggesting they are less conserved. Only one of these nine unknown contigs, contig Llo.58, assigned to LPRV2, had a complete ORF that was predicted to encode a 361 amino acid protein containing two putative domains (Supplementary Figure S6). The first domain is a Zn-dependent metallopeptidase from the Astacin superfamily found in digestive enzymes in both invertebrates and vertebrates (45,46). The second domain is a Peritrophin-A found in chitin-binding proteins that includes peritrophic matrix proteins of insect chitinases also found in baculoviruses (47). Thus, contig Llo.58 could encode a protein involved in the interaction between LPRV2 and sandflies since viruses commonly hijack and repurpose cellular proteins to their own advantage. Genes involved in host-pathogen interactions tend to be more divergent among viruses. Importantly, Llo.58 was not detected by similarity searches against reference databases and would not have been classified as viral based solely on domain prediction since these could also be found in cellular proteins. Thus, analysis of the small RNA size profile identified 23 unknown contigs representing additional segments of LPRV1 and LPRV2 genomes that have no similarity to known sequences in reference databases.

Unknown contigs found in *Drosophila* libraries were grouped in two separate clusters. Cluster 4 included five unknown contigs that showed high similarity to the cluster containing both DUV and DCV (Figure 4A). Cluster 5 contained another 14 unknown contigs that showed similarity to the profile of DUV and DCV albeit at lower correlation than cluster 4 (Figure 4A). Since the full genome sequence of DCV is known, these unknown contigs in the two separate clusters most likely represent different contigs from DUV. Indeed, we only identified two DUV contigs corresponding to the viral RdRP, which represents a small percentage of the full genome. In agreement with this hypothesis, these contigs were only found in the *Drosophila* library where DUV was identified (data not shown).

In *Aedes* libraries, 24 out of 27 unknown contigs were grouped in six different clusters (7, 8, 9, 10, 11 and 12) that showed high correlation to each other and a small RNA size profile consistent with mosquito piRNAs (27–28 nt peak in the size profile) (Figure 4A) (20,48). Accordingly, small RNAs derived from these contigs showed enrichment for U at position 1 and A at position 10, typical of insect piRNAs but no substantial 21 nt size peak nor symmetric coverage of both strands (data not shown). Thus, these sequences are most likely derived from repetitive regions that generate abundant piRNAs but are still absent from the current version of the *A. aegypti* genome.

### The small RNA profile can provide information about virus biology

The pattern of small RNAs generated by the host response depends on virus characteristics such as genome structure, tissue tropism or strategy of replication. Thus, besides identifying viral contigs, the small RNA size profile may also provide specific information on the biology of each virus. For example, RNA viruses tend to have a very homogenous small RNA coverage of the viral genome while DNA viruses show clear hotspots of small RNAs (18,22,23). All viral contigs described here were derived from RNA viruses and mostly had homogeneous small RNA coverage.

**Figure 5.**
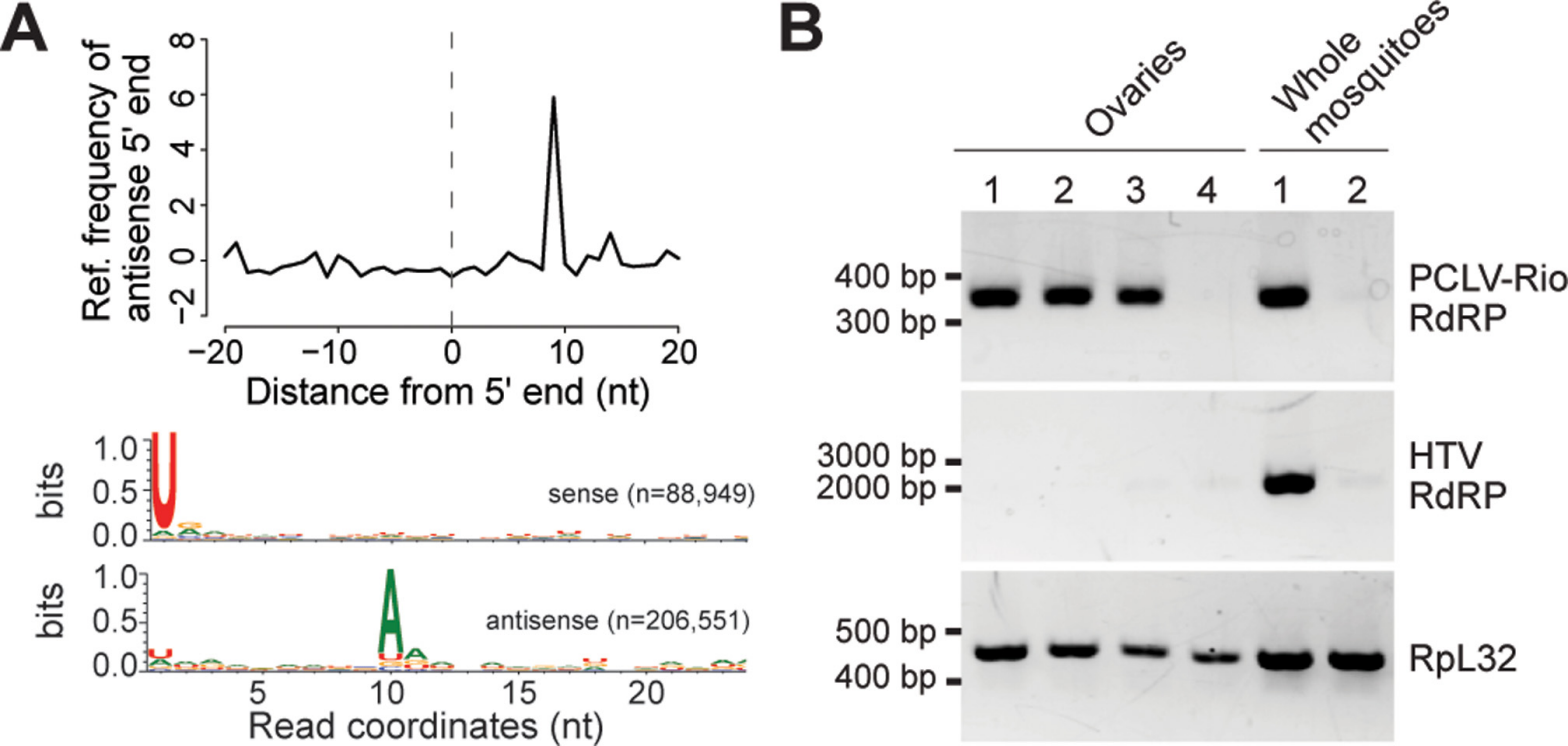
The presence of virus-derived piRNAs with a ping-pong signature is indicative of ovary infection. (**A**) About 24–29 nt small RNAs derived from PCLV show a 10 nt overlap between sense and antisense strands and U enrichment at position 1 and A enrichment at position 10 consistent with piRNAs generated by the ping-pong amplification mechanism found in the insect germline. (**B**) Both PCLV and HTV are detected in individual mosquitoes but only PCLV is present in ovaries as determined by RT-PCR. Results are representative of eight ovaries of individual mosquitoes that were analysed. The endogenous gene *Rpl32* was used as control for the RT-PCR.

We also noticed that HTV and PCLV showed very distinct small RNA size profiles despite being sometimes found in the same mosquitoes (Figures 2D and 3A). The profile of HTV showed a clear 21-nt peak size consistent with production of siRNAs. In contrast, the profile of PCLV showed two separate peaks of 21 and 24–29 nt, consistent with small RNAs generated by both siRNA and piRNA pathways. Indeed, 24–29 nt small RNAs derived from PCLV showed enrichment for U at position 1 and A at position 10, typical of sense and antisense insect piRNAs, respectively (Figure 5A). The insect piRNA pathway is mostly active in the germline where two mechanisms of small RNA biogenesis may occur (49). Primary sense piRNAs are generated by endonucleolytic processing of precursor transcripts while secondary piRNAs are produced by an amplification loop referred to as the ping-pong mechanism. We observed that 24–29 nt small RNAs derived from PCLV showed 10-nt overlap between sense and antisense RNAs consistent with the ping-pong amplification mechanism (Figure 5A) (50,51). These results suggest that PCLV induces the production of piRNAs by this mechanism when infecting the insect germline. In agreement with this hypothesis, we observed 75% prevalence for PCLV in ovaries of individual mosquitoes (Figure 5B). In contrast, HTV was not found in ovaries consistent with the fact it does not generate piRNAs (Figure 5B). Thus, the presence of a clear piRNA signature in the small RNA profile could help infer tissue tropism for the insect germline.

The lack of clear peaks in the size distribution of small RNAs may suggest inhibition of RNAi pathways such as reported for *Flock House virus* (FHV). Indeed, the B2 protein encoded by FHV is a powerful suppressor of silencing that blocks the RNAi pathway (52). Interestingly, ORF 2 in RNA 1 from the LPNV genome is predicted to encode a protein with similarity to the FHV B2 protein that could act as a suppressor of silencing (Supplementary Figures S6 and S7). Thus, the broad small RNA size profile with no clear peaks observed for LPNV could suggest inhibition of RNAi pathways as a strategy of replication (Figure 3A). A broad size profile and strong preference for small RNAs generated from the positive strand of the viral genome was also observed for DCV and DUV in infected fruit flies (Figure 3 and Supplementary Figure S1). Since the DCV-1A gene encodes a potent suppressor of the siRNA pathway (53), this suggests that DUV may also be capable of suppressing RNAi in infected flies.

### Virus detection in published insect small RNA libraries

**Figure 6.**
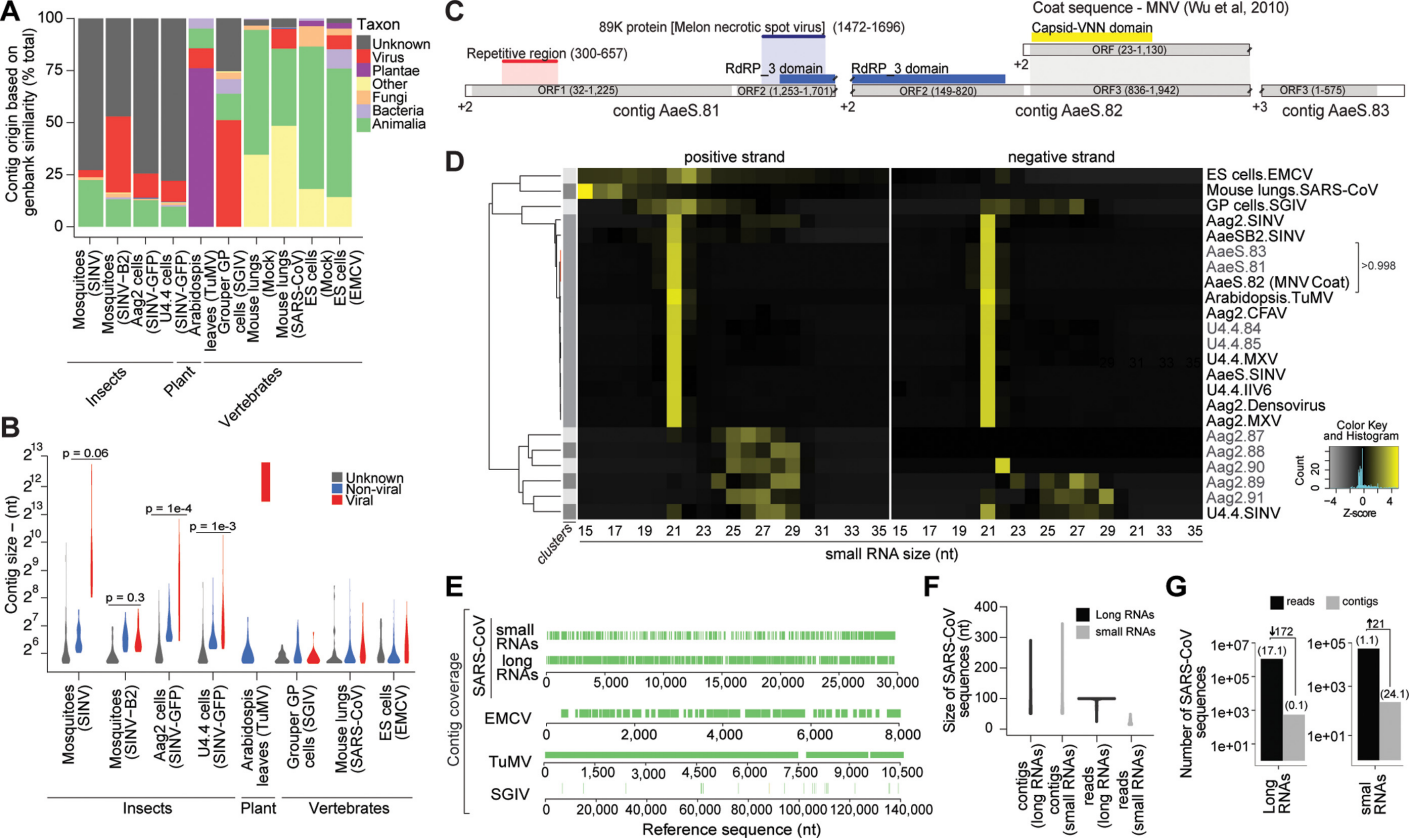
Virus detection based on large-scale sequencing of small RNAs is applicable to animals and plants. (**A**) Percentage of contigs assembled from published small RNA libraries from insects, plants and vertebrate animals with significant similarity against reference sequences. The origin of contigs is classified by taxon and includes unknown sequences. (**B**) Size distribution of contigs corresponding to viral (red), non-viral (blue) or unknown sequences (grey) in each library. *P*-values for the difference between contig sizes are indicated (Student *t*-test). (**C**) Hypothetical genome organization of MNV based on ORF and small RNA analysis of contigs AaeS.81, AaeS.82 and AaeS.83 identified in this study. (**D**) Hierarchical clustering of viral and unknown contig sequences assembled in published libraries. Clustering was based on Pearson correlation of the small RNA size profile shown as a heatmap. A single cluster with more than one contig is indicated on the left vertical bar as defined by correlation above 0.8. A sub-cluster highlighted in red contains small RNA profiles of three contigs that show Pearson correlation above 0.998. (**E**) Coverage of SARS-CoV, EMCV, TuMV and SGIV genomes by contigs assembled in RNA libraries from mouse lungs, ES cells, *Arabidopsis* and fish GP cells, respectively. (**F**) Size distribution of contigs and raw sequenced reads derived from SARS-CoV in long (black) or small (grey) RNA libraries from infected mouse lungs. (**G**) Number of raw reads and contigs sequences derived from viruses in long and small RNA libraries prepared from SARS-CoV infected mouse lungs. The number above bars indicates the percentage of viral reads and contigs sequences relative to the total. Fold enrichment or depletion of virus sequences comparing contigs to raw reads is shown.

We decided to validate our strategy by analysing four published insect small RNA libraries constructed from adult mosquitoes and cell lines infected with SINV (Supplementary Table S2) (21,25). Sequence similarity searches showed that viral sequences represented 10.9% of contigs assembled from these datasets (Figure 6A). Size difference between viral and non-viral contigs was significant in most cases with the exception of mosquitoes infected with a recombinant SINV encoding the FHV B2 protein that almost completely blocks the RNAi pathway (Figure 6B) (54). Nevertheless, SINV sequences were detected among viral contigs in all libraries including the one where the RNAi was inhibited. This suggests that virus-derived small RNAs produced by host RNAi pathways are important but not essential for the assembly of viral contigs. Our approach also detected the presence of several contigs derived from viruses that were not reported at the time of first publication. We detected contigs derived from *A. aegypti densovirus 2* (AaDV2), *Mosquitoe X virus* (MXV) and *Cell fusion agent virus* (CFAV) in Aag2 cells, MXV and *Insect Iridescent virus- 6* (IIV6) in U4.4 cells and *Mosquito nodavirus* (MNV) in adult mosquitoes (Supplementary Table S3). Notably, a 1130 nt sequence corresponding to MNV was originally identified by another small RNA-based analysis pipeline in the library from adult mosquitoes (15). Using the same dataset, our strategy assembled a contig of 1994 nt (AaeS.82) that extended the original published MNV sequence of 1130 nt (Figure 6C). This 1994 nt MNV sequence contains the original ORF encoding the capsid protein and an additional incomplete ORF predicted to encode a protein with an RdRP_3 domain (PF00998) (Figure 6C). In addition, we detected a viral contig of 1702 nt (AaeS.81) that showed significant similarity to *Melon necrotic spot virus*, a member of *Tombusviridae* family (Supplementary Table S3). Contig AaeS.81 has one complete ORF of 397 aminoacids and a second incomplete ORF that contains a RdRP_3 conserved domain (PF00998), the same domain found in the MNV contig (Figure 6C). The small RNA size profile of contig AaeS.81 and MNV (AaeS.82) are very similar and showed correlation above 0.998 (Figure 6D). These results suggest that the 1994 nt MNV sequence and contig AaeS.81 could represent different fragments of the same viral genome (Figure 6C). In agreement with this hypothesis, contig AaeS.81 and MNV (AaeS.82) were only found in the same library prepared from adult mosquitoes infected with SINV.

In these published libraries, our pipeline also assembled a total of 1673 unknown contigs. Small RNA profiles were analysed for eight unknown contigs longer than the N50 observed for viral contigs (208 nt). Regarding the small RNA size profile, most viral contigs identified in published insect datasets were grouped in a single large cluster showing a 21 nt peak size consistent with typical siRNAs (Figure 6D). The lack of diversity in the small RNA profile can be explained by the higher homogeneity of these samples that are mostly derived from mosquitoes. Nevertheless, one unknown contig of 709 nt, contig AaeS.83, showed small RNA size profile similar to MNV (AaeS.82) and AaeS.81 and were grouped in the same cluster with correlation above 0.998 (Figure 6D). It is tempting to speculate that contig AaeS.83 might represent another missing piece of the MNV genome together with AaeS.81 (as suggested in Figure 6C).

We also identified two unknown contigs of 390 and 363 nt in U4.4 cells, U4.4.84 and U4.4.85, that showed a size profile similar to several viruses grouped together (Figure 6D). Contig U4.4.84 is predicted to encode two incomplete ORFs one of which shows limited similarity to *Megavirus terra 1* (Supplementary Figure S8A). High correlation of the small RNA size profile suggests U4.4.84 and U4.4.85 have the same origin. We also found small RNAs derived from contigs U4.4.84 and U4.4.85 in the small library prepared from Aag2 cells in the same laboratory as U4.4 cells (Supplementary Figure S8B). These observations could suggest an infectious virus that contaminated both cell cultures since small RNAs derived from contigs U4.4.84 and U4.4.85 were not observed in Aag2 cells from our own laboratory (data not shown). Another five unknown contigs were assembled in the library from Aag2 cells but showed small RNA profiles consistent with piRNAs suggesting these might represent repetitive regions absent from the mosquito genome.

### Small RNAs allow efficient virus detection in plants and vertebrate animals

Virus detection utilizing small RNAs has been applied to insects and plants but not to vertebrates to the best of our knowledge (14–17). In order to further test our strategy, we analysed small RNA libraries prepared from *Arabidopsis thaliana* leaves infected with *Turnip mosaic virus* (TuMV), grouper fish GP cells infected with *Singapore grouper iridovirus* (SGIV), mouse lungs infected with *Severe acute respiratory syndrome coronavirus* (SARS-CoV) and mouse embryonic stem cells infected with *Encephalomyocarditis virus* (EMCV) (55–58). Samples infected with known viruses were chosen to provide proof-of-concept for detection. Although the small RNA profile was diverse, contigs corresponding to each virus were efficiently and specifically detected by sequence-based comparisons in the respective infected samples (Figure 6D and E). Notably, viral sequences assembled in *Arabidopsis* were among the largest contigs assembled in all libraries we analysed in this study (Figure 6B). This is most likely the result of highly efficient production of virus-derived small RNAs by the plant RNAi pathway that favours assembly of long viral contigs (Figure 6E) (16,59).

In contrast to *Arabidopsis*, viral contigs assembled in mouse and fish libraries were among the shortest (Figure 6B). This suggests that viral contig assembly from small RNAs in vertebrate animals is not as efficient as in insects and plants. Nevertheless, contigs were assembled and allowed identification of viruses in all fish and mouse small RNA libraries. In fish GP cells, the smaller size of SGIV contigs could be partially explained by restricted generation of dsRNA during the replication cycle of dsDNA viruses (23). Results obtained with mouse small RNA libraries suggest that activation of RNAi is not essential to allow assembly of viral contigs. EMCV contigs were detected in ES cells where the RNAi pathway is activated (56). In contrast, SARS-CoV contigs were assembled in mouse lungs from small RNAs that were most likely generated by other RNAses (Figure 6F) (57). Notably, RNase L is an important antiviral factor that can degrade viral RNAs in mammalian cells independently of RNAi (28). The size distribution of viral contigs and number of virus-derived small RNAs was similar for EMCV and SARS-CoV (Figure 6B and E). Thus, small RNAs generated by the RNAi pathway or resulting from degradation by other RNases both allowed similar assembly of viral contigs in mouse samples.

We also directly compared virus identification from different RNA fractions prepared from mouse lungs infected with SARS-CoV (57,60). SARS-CoV contigs assembled from long and small RNAs had a similar size distribution despite the larger read size and numbers observed in the library prepared from long RNAs (Figure 6E and F). Small RNA libraries showed more than 20-fold enrichment of viral sequences among contigs when compared to raw reads (Figure 6G). In contrast, there is a 172-fold decrease in the percentage of viral sequences detected in assembled contigs compared to raw reads from long RNA libraries (Figure 6G). Thus, contig assembly from small RNAs favours assembly of SARS-CoV sequences compared to long RNAs even though no clear RNAi response is observed in mouse lungs. These preliminary results suggest that small RNAs show enrichment for viral sequences and can be used to assemble contigs not only in insects and plants but also mammals.

We also observed that very few contigs assembled by our pipeline in small RNA libraries from mice, fish and plants were unknown sequences (Figure 6A and B). Thus, our strategy to use the small RNA size profile to characterize unknown contigs could not be properly tested in these samples. Further testing is required to evaluate the application of our pattern-based approach to vertebrates and also plants.

## DISCUSSION

In this study we describe a powerful approach based on small RNAs that allows for successful identification of viruses without any prior information about their presence. The majority of the viruses we identified potentially represent new species, illustrating the power of our strategy. Importantly, our results strongly indicate that virus identification from small RNAs provides four notable advantages compared to other metagenomic strategies. Firstly, preparation of small RNA libraries requires little sample manipulation and no column filtration steps before RNA extraction. This minimizes the chance of sample contamination or bias that can affect virus discovery by metagenomic studies (8). Secondly, we demonstrate that large scale sequencing of small RNAs optimizes the detection of viruses since these are naturally enriched for viral sequences and favour assembly of longer contigs compared to long RNAs. This is likely a result of the mechanism of small RNA biogenesis by host antiviral pathways that seem to efficiently generate large amounts of overlapping virus-derived small RNAs. Thirdly, we show that the small RNA size profile can help identify and characterize potential novel viral sequences for which we would otherwise have no other information. Indeed, large-scale sequencing projects currently face limitations due to the amount of sequences without known relatives in reference databases (10). We observed that small RNA size profiles are quite specific, and show that pattern similarities can be used to identify novel viral sequences. Using this approach we characterized novel viral segments of three viruses described in this study, HTV, LPRV1 and LPRV2. Fourthly, we show that the small RNA profile could help infer specific features of virus biology such as genome structure, tissue tropism and replication strategies. Indeed, based on the presence of a signature observed for activation of the piRNA pathway in the insect germline, we demonstrated that PCLV but not HTV is found in mosquito ovaries.

A large part of our strategy was based on the diversity of virus-derived small RNA profiles observed in infected insects. Although virus-derived small RNAs profiles can be very heterogeneous in infected insects, only the production of 21 nt long virus-derived siRNAs has classically been considered a hallmark of antiviral immunity (15,17,18,20–23,61). Our high-throughput analysis of three insect species infected with 10 different viruses shows a diversity of virus-derived small RNAs profiles that do not reflect technical differences in sample preparation, processing or analysis. Rather, these distinct profiles of virus-derived small RNA profiles seem to reflect divergent strategies of viral replication and host-specific antiviral responses.

Using our small RNA-based approach we characterized the virome of laboratory stocks of fruit flies and wild populations of two vector insects, mosquitoes and sandflies. These included six novel viruses and a strain of PCLV previously described in mosquitoes from Thailand. Of particular significance, we identified viruses belonging to viral families (e.g. *Bunyaviridae* and *Reoviridae*) that include several mammalian pathogens. Future studies should evaluate the presence of these viruses in wild mosquito and sandfly populations in Brazil as a potential threat for humans and livestock. In addition, these viruses could affect the ability of vector insects to carry other human pathogens such as *Dengue virus* and *Leishmania*, naturally transmitted by mosquitoes and sandflies, respectively. Together our results indicate that sequencing of small RNAs is a powerful virus surveillance strategy in research laboratories as well as natural settings.

Our small RNA based strategy was also successful in characterizing viruses in published small RNA datasets from plants, fish and mammals in addition to insects. In the case of mouse samples, enrichment of viral sequences in the small RNA fraction was observed even in the absence of activation of the RNAi pathway. Thus, efficient production of virus-derived small RNAs might be a broad phenomenon that can be further explored for virus detection. Indeed, multiple mammalian antiviral pathways, including RNAi and RNase L, can generate small RNAs during viral infection (28,56). However, viral contigs assembled from small RNAs were all identified by sequence similarity searches against reference databases. Thus, more extensive analyses are still required in order to evaluate whether our small RNA profile based approach can have broad applications to plants and animals.

## ACCESSION NUMBERS

Datasets were deposited on the Small Read Archive of the National Center for Biotechnology Information under accession numbers described in Supplementary Table S2. Viral sequences described in Table 1 were deposited in Genbank under accession numbers KR003784–KR003824.

## SUPPLEMENTARY DATA

Supplementary Data are available at NAR online.

SUPPLEMENTARY DATA
