# Sequence-independent characterization of viruses based on the pattern of viral small RNAs produced by the host

**DOI:** 10.1093/nar/gkw044

**Published:** 2016-01-21

**Authors:** Eric Roberto Guimarães Rocha Aguiar, Roenick Proveti Olmo, Simona Paro, Flavia Viana Ferreira, Isaque João da Silva de Faria, Yaovi Mathias Honore Todjro, Francisco Pereira Lobo, Erna Geessien Kroon, Carine Meignin, Derek Gatherer, Jean-Luc Imler, João Trindade Marques

**Affiliations:** 1 Department of Biochemistry and Immunology, Instituto de Ciências Biológicas, Universidade Federal de Minas Gerais, Belo Horizonte, Minas Gerais, CEP 30270–901, Brazil; 2 CNRS-UPR9022, Institut de Biologie Moléculaire et Cellulaire, 67084 Strasbourg Cedex, France; 3 Department of Microbiology, Instituto de Ciências Biológicas, Universidade Federal de Minas Gerais, Belo Horizonte, Minas Gerais, CEP 30270–901, Brazil; 4 Laboratório Multiusuário de Bioinformática, Embrapa Informática Agropecuária, Campinas, São Paulo, CEP 13083–886, Brazil; 5 Faculté des Sciences de la Vie, Université de Strasbourg, 67083 Strasbourg Cedex, France; 6 Division of Biomedical and Life Sciences, Faculty of Health and Medicine, Lancaster University, Lancaster, Lancashire, LA1 4YQ, United Kingdom; 7 Institut d'Etudes Avancées de l'Université de Strasbourg (USIAS), 67084 Strasbourg Cedex, France

*Nucl. Acids Res*. 43 (13): 6191–6206. doi: 10.1093/nar/gkv587

The authors wish to make the following corrections to their article:

1. In the section ‘**The small RNA profile can provide information about virus biology**’ of RESULTS, the order of the words sense and antisense was mistakenly inverted. The fourth sentence of the second paragraph on this section of the original manuscript reads:
  In contrast, the profile of PCLV showed two separate peaks of 21 and 24–29 nt, consistent with small RNAs generated by both siRNA and piRNA pathways. Indeed, 24–29 nt small RNAs derived from PCLV showed enrichment for U at position 1 and A at position 10, typical of **sense and antisense** insect piRNAs, respectively (Figure 5A).
  The correct text should read as below:
  In contrast, the profile of PCLV showed two separate peaks of 21 and 24–29 nt, consistent with small RNAs generated by both siRNA and piRNA pathways. Indeed, 24–29 nt small RNAs derived from PCLV showed enrichment for U at position 1 and A at position 10, typical of **antisense and sense** insect piRNAs, respectively (Figure 5A).
2. A similar mistake was committed in Figure 5.
  In panel A of Figure 5, antisense and sense labels in panel A were inverted. The order of the words sense and antisense was also inverted in the legend of Figure 5. The correct Figure and legend are provided below.

**Figure 5.**
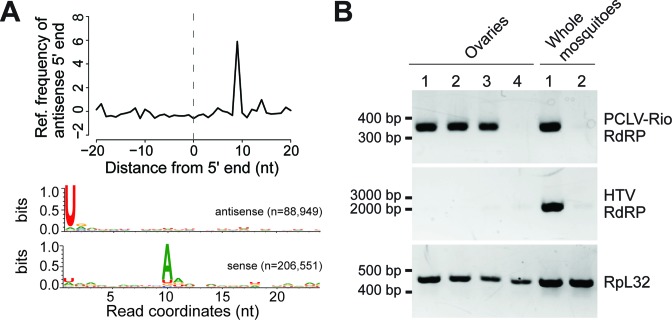
The presence of virus-derived piRNAs with a ping-pong signature is indicative of ovary infection. (A) 24–29 nt small RNAs derived from PCLV show a 10 nt overlap between **antisense and sense** strands and U enrichment at position 1 and A enrichment at position 10 consistent with piRNAs generated by the ping-pong amplification mechanism found in the insect germline. (B) Both PCLV and HTV are detected in individual mosquitoes but only PCLV is present in ovaries as determined by RT-PCR. Results are representative of 8 ovaries of individual mosquitoes that were analyzed. The endogenous gene Rpl32 was used as control for the RT-PCR.

The results and conclusion of the article are not affected and remain valid. The authors apologize to the readers for the inconvenience caused.

