# Supplementary material for: Sequence-independent characterization of viruses based on the pattern of viral small RNAs produced by the host
Source: Nucleic Acids Res. 2015 Jun 3;43(13):6191–206. doi: 10.1093/nar/gkv587 (PMC4513865; doi:10.1093/nar/gkv587)
Supplement: SUPPLEMENTARY DATA [file supp_43_13_6191__index.html]

Sequence-independent characterization of viruses based on the pattern of viral small RNAs produced by the host — Sequence-independent characterization of viruses based on the pattern of viral small RNAs produced by the host — SUPPLEMENTARY DATA 

# Sequence-independent characterization of viruses based on the pattern of viral small RNAs produced by the host

## SUPPLEMENTARY DATA

- SUPPLEMENTARY DATA
