# Supplementary material for: Sequence-independent characterization of viruses based on the pattern of viral small RNAs produced by the host
Source: Nucleic Acids Res. 2015 Jun 3;43(13):6191–206. doi: 10.1093/nar/gkv587 (PMC4513865; doi:10.1093/nar/gkv587)
Supplement: SUPPLEMENTARY DATA [file supp_gkv587_nar-00840-z-2015-File009.pdf]

SUPPLEMENTARY DATA

Sequence-independent characterization of viruses based on the pattern of viral small RNAs produced by the host

Eric Roberto Guimaraes Rocha Aguiar[1,2], Roenick Proveti Olmo[1,2], Simona Paro[2], Flavia Viana Ferreira[3], Isaque João da Silva de Faria[1], Yaovi Mathias Honore Todjro[1], Francisco Pereira Lobo[4], Erna Geessien Kroon[3], Carine Meignin[2,5], Derek Gatherer[6], Jean-Luc Imler[2,5,7], Joao Trindade Marques[1,*]

[1]Department of Biochemistry and Immunology, Instituto de Ciências Biológicas, Universidade Federal de Minas Gerais, Belo Horizonte, Minas Gerais, CEP 30270-901, Brazil

[2]CNRS-UPR9022, Institut de Biologie Moléculaire et Cellulaire, 67084 Strasbourg Cedex, France

[3]Department of Microbiology, Instituto de Ciências Biológicas, Universidade Federal de Minas Gerais, Belo Horizonte, Minas Gerais, CEP 30270-901, Brazil

[4]Laboratório Multiusuário de Bioinformática, Embrapa Informática Agropecuária, Campinas, São Paulo, CEP 13083-886, Brazil

[5]Faculté des Sciences de la Vie, Université de Strasbourg, 67083 Strasbourg Cedex, France

[6]Division of Biomedical and Life Sciences, Faculty of Health and Medicine, Lancaster University, Lancaster, Lancashire, LA1 4YQ, United Kingdom

[7]Institut d'Etudes Avancées de l'Université de Strasbourg (USIAS), 67084 Strasbourg Cedex, France

Contents **Page**

Supplementary Methods
Phylogenetic and Dinucleotide frequency analyses 2

Supplementary Tables
Supplementary Table S1. Oligonucleotide primers used for PCR analysis 3
Supplementary Table S2. Overview of libraries analyzed in this study 4
Supplementary Table S3. Summary of viruses identified in published small RNA libraries 5
Supplementary Table S4. Reference viral sequences used for phylogenetic and dinucleotide frequency analyses 6

Supplementary Figures
Supplementary Figure S1. Virus-derived small RNA can be assembled into long contig sequences 7
Supplementary Figure S2. Unknown contigs classify small RNA libraries in a host-specific manner 8
Supplementary Figure S3. Viruses identified by small RNA sequencing belong to diverse viral families according to phylogenetic and dinucleotide analyses 9
Supplementary Figure S4. Small RNA size profile of representative contigs derived from Animalia, Bacteria and Fungi 11
Supplementary Figure S5. Polymerase and capsid segments from HTV and DAV show similarity to different viral families 12
Supplementary Figure S6. ORF organization and domain analysis of viral sequences identified by our strategy 13
Supplementary Figure S7. Analysis of B2-like protein from LPNV 14
Supplementary Figure S8. ORF structure and small RNA profile of unknown viral sequences identified in small RNA libraries from U4.4 cells 15

Supplementary References 16

SUPPLEMENTARY METHODS

Phylogenetic and dinucleotide frequency analyses. Nucleotide or protein sequences were chosen based on BLAST similarity and were aligned using Muscle (62), implemented in MEGA (63). The best substitution model was estimated using maximum likelihood (ML) methods, and ML trees constructed using 100 bootstrap replicates. The consensus trees were midpoint–rooted. Where the bootstrap confidence of a node exceeded 70%, the bootstrap values are indicated on the trees. Nucleotide sequence ML trees were drawn in MEGA using the Tamura 3-parameter substitution model ($T92+\Gamma$) (64). Protein sequence ML trees were drawn in MEGA using the Poisson substitution model (in one case, the WAG substitution model was used as indicated) (65,66). Dinucleotide frequency analysis in each contig or reference sequence was calculated and results clustered based on Spearman's rho correlation to build dendograms essentially as described (67). Organism list, acronyms and identifiers for each database are shown in **Supplementary Table S4.**

Supplementary Table S1. Oligonucleotide primers used for PCR analysis.

Target	Tm (^{o}C)	Forward	Reverse
**Viruses**			
PCLV	55	CTATTATTGGCACCCCTGAA	CCAGATCCTAGCATTGGTTT
HTV	55	GTATACGCGTTGGTGAGTAT	CCGACTCAGCATAATTACGA
Aae.92	55	GTCTGATTTGCCCAACTCTA	CAGCATCGCAGGTTATAGTA
LPRV1	55	CCATGATCCAGCAATTCAAC	GTGCACACATATCATAAGCG
LPRV2	55	CTGGAAGATCAATGGTGTGA	TAATGGCGATGGACGATAAG
LPNV	55	GTGTTAATTGTGTGCGTTCC	GGTGACTCAATCAATGAACG
DUV	55	GAACTATCGCACCGTTTAAC	GTTGTGTCGTGTCTAGAAGT
DRV	55	GTGTGGTCTACATGTCAAGT	GGTAACAGCGTGTACCATAT
**LPRV1 Segments**			
3330/3331	55	ACACGTCGTTAATACCTCAG	GTTGTGAAGTAACTGGCAAC
3332/3333	55	AAGTAAACCCAGACCACATC	GTGTAGAGTATATGCGTGCA
3310/3311	55	ATTCCAGTCAGCGTAAAGTT	AGGTGTGATGGCATTGTAAT
3312/3313	55	TAATAGTTGTAGCCATGGCC	TCTCCACAGAGCAATCAATC
**LPRV2 Segments**			
3336/3337	55	TGCTACTCTAGTTCTCGTCA	CCATCTAAGTGTCAGCGTTA
3338/3339	55	AATATAGCCTATGCGACGAC	ACCACATGTATAATCGACGG
3314/3315	55	TTCCTGACGGGTAGACATAT	GAGTGCAAGCATATGACAAC
3318/3319	55	GGTATAACACGGTTTCCTGT	TACTACACTGCGGCTAGTT
3316/3317	55	CATGCAAGGAACATGATGTC	TATGTCAATGTGCGCATCTA

Supplementary Table S2. Overview of RNA libraries analyzed in this study.

Library	Number of pooled individuals	SRA ID	Artificial infection	Total number of reads	Number of mapped reads on host	Number of processed reads	Number of contigs	N50 (nt)	Size of largest contig (nt)	Number of contigs hit virus
**Libraries sequenced in this study**										
Drosophila melanogaster										
Dme_1	6	SRR1803381	SINV	4,234,079	3,194,745	596,761	327	79	4,765	41
Dme_2	6	SRR1803382	DCV	15,786,440	11,483,909	2,018,666	343	137	3,496	5
Dme_3	6	SRR1803383	VSV	24,474,261	21,701,073	1,039,581	171	288	3,482	37
***Aedes aegypti* – small RNA**										
Aae_1	6	SRR1803377	none	9,081,151	8,076,206	891,983	1,686	67	2,301	16
Aae_2	6	SRR1803378	none	12,183,902	10,827,108	999,843	1,658	66	1,611	17
Aae_3	6	SRR1803379	none	9,253,941	8,010,814	1,158,379	2,722	68	5,122	12
***Aedes aegypti* – Long RNA**										
Aae_1	6	SRR1813817	none	39,488,681	35,767,399	3,721,282	295,760	136	2,070	12
Aae_2	6	SRR1813823	none	57,584,234	52,130,913	5,453,321	358,323	136	1,988	17
Aae_3	6	SRR1813824	none	62,302,651	56,383,441	5,919,210	357,264	138	2,334	9
Lutzomya longipalpis										
Llo_1	8	SRR1803384	none	12,297,884	10,852,586	483,139	1,207	69	1,345	14
Llo_2	7	SRR1803385	none	9,463,241	8,162,975	449,4327	2,151	63	980	12
Llo_3	7	SRR1803386	none	8,109,613	7,285,842	659,215	1,541	78	1,113	31
**Published small RNA libraries**										
U4.4 cells	-	SRR389184	SINV-GFP	27,997,328	20,467,497	6,689,669	1,086	72	1,273	95
Aag2 cells	-	SRR389187	SINV-GFP	35,569,242	21,247,368	9,316,546	763	87	1,874	79
Mosquitoes	-	SRR400496	SINV	4,238,851	2,980,359	1,300,122	226	197	7,361	9
Mosquitoes	-	SRR400497	SINV-NoVB2	3,522,010	2,874,099	689,541	219	74	201	67
Arabidopsis leaves	-	SRR1561607	TuMV	15,841,206	6,269,276	6,532,498	75	2,896	7,706	2
Grouper GP cells	-	SRR096455	SGIV	7,246,099	4,742,849	936,104	147	64	154	22
Mouse lungs	-	SRX377856	SARS-CoV	44,436,105	37,200,539	6,835,566	528,894	36	291	0
Mouse lungs	-	SRR452408	SARS-CoV	22,665,163	16,979,135	2,914,479	924	66	429	140
Mouse lungs	-	SRR452409	none	24,311,834	17,067,263	3,123,142	757	57	212	0
Mouse ES cells	-	SRR640604	EMCV	64,913,697	34,379,621	13,995,769	898	62	255	69
Mouse ES cells	-	SRR640602	none	56,784,360	33,088,484	12,742,698	867	61	293	0

Supplementary Table S3. Summary of viruses identified in published small RNA libraries.

SRA ID/sample	Virus family	Virus	Origin of viral sequence	Strategy	Largest contig size (nt)	Number of hits	Best hit	E-value	Accession number
**SRR389184/ *Aedes albopictus* U4.4 cells + SINV-GFP**	*Togaviridae*	*Sindbis virus*	experimental infection	blastx	1,128	38	nonstructural polyprotein [Sindbis virus]	0.0	AAA96975.1
	Iridoviridae	*Insect iridescent virus-6*	unknown	blastx	73	1	IIV6 genome	1,00E-07	AF303741.1
	Birnaviridae	*Mosquitoe X virus*	unknown	blastx	195	54	polyprotein [Mosquitoe x virus]	4,00E-39	AFU34333.1
	unkonwn	Unknown (contig U4.4.84)	unknown	pattern-based	390	1	-	-	-
	unknown	Unknown (contig U4.4.85)	unknown	pattern-based	363	1	-	-	-
**SRR389187/ *Aedes aegypti* Aag2 cells + SINV-GFP**	*Flaviviridae*	*Cell fusing agent virus*	unknown	blastx	203	47	polyprotein [Cell fusing agent virus]	3,00E-11	P33515.1
	Birnaviridae	*Mosquitoe X virus*	unknown	blastx	87	10	putative VP1 [Mosquitoe x virus]	2,00E-10	AFU34334.1
	Togaviridae	*Sindbis virus*	experimental infection	blastx	1,874	18	polyprotein [Sindbis virus]	0.0	ACU25468.1
	Parvoviridae	*Aedes aegypti densovirus 2*	unknown	blastx	52	1	Aedes aegypti densovirus 2	5,00E-08	NC_012636.1
	Togaviridae	*Sindbis virus*	experimental infection	blastx	7,361	2	nonstructural polyprotein [Sindbis virus]	0.0	AAA96974.1
**SRR400496/ *Aedes aegypti* + SINV**	*Nodaviridae*	*Mosquito nodavirus*	unknown	blastx	1,943	2	coat protein [Mosquito nodavirus MNV-1]	1,00E-161	ACY74430.1
		Mosquito nodavirus (contig AaeS.82)	unknown	blastx	1,729*	2	p89 [Melon necrotic spot virus]	8,00E-09	AGO36278.1
		Mosquito nodavirus (contig AaeS.83)	unknown	pattern-based	709	1	-	-	-
**SRR400497/ *Aedes aegypti* + SINV-B2**	*Togaviridae*	*Sindbis virus*	experimental infection	blastx	169	65	polyprotein [Sindbis virus]	3,00E-32	BAH70330.1
**SRR1561607 / *Arabidopsis* + TuMV**	*Potyviridae*	*Turnip mosaic virus*	experimental infection	blastn	7,771	3	polyprotein [Reporter vector pCBTuMV-GFP]	0	ABK27329.1
**SRR096455 / Grouper GP cells +SGIV**	*Iridoviridae*	*Singapore grouper iridovirus*	experimental infection	blastn	112	22	Singapore grouper iridovirus, complete genome	1,00E-17	AY521625.1
**SRR452408 / Mouse lungs + SARS-CoV**	*Coronaviridae*	*Severe acute respiratory syndrome coronavirus*	experimental infection	blastn	335	137	SARS coronavirus, complete genome	8,00E-90	JN854286.1
**SRR640604 / Mouse ES cells + EMCV**	*Picornaviridae*	*Encephalomyocarditis virus*	experimental infection	blastn	243	56	Polyprotein [Encephalomyocarditis vírus]	2E-49	ACI47517.1

Supplementary Table S4. Reference viral sequences used for phylogenetic and dinucleotide frequency analyses.

Family	Genus	Virus	Accession number (Uniprot or GenBank)
Bunyaviridae	*Hantavirus*	*Hantaan virus (HanV)*	P23456
		Seoul virus Hantavirus (SeV)	P27314
		Tula virus (Tula)	AJ005637.1
	Nairovirus	*Crimean-Congo hemorrhagic fever (CrCHFV)*	Q6TQR6
		Dugbe virus isolate ArD44313 (DuV)	Q66431
	Orthobunyavirus	*Bunyamwera virus (BunV)*	P20470
		La crosse (LCV)	Q8JPR2
	Phlebovirus	*Rift Valley fever virus (RVFV)*	P27316
		Uukuniemi virus (strain S23) (UkV)	P33453
	Tospovirus	*Tomato spotted (strain Brazilian Br-01)*	P28976
		Melon yellow spot virus (MYSV)	AB061774.1
		Bean necrotic mosaic virus (BNMV)	NC_018070.1
		Groundnut bud necrosis virus (GBNV)	NC_003614.1
	Tenuivirus	*Rice stripe virus (RSTV)*	Q85431
		Rice grassy (RGTV)	NC_002323.1
	Unclassified	*Phasi Charoen-like virus (PCLV)*	KM001085.1
Reoviridae	*Aquareovirus*	*Aquareovirus C (isolate Golden shiner/USA/GSRV/1977)*	Q8JU61
		Aquareovirus G (isolate American grass carp/USA/PB01-155/)	B2BNE0
		Aquareovirus A (isolate Chum salmon/Japan/CSRV/1981)	Q8VA42
	Coltivirus	*Colorado tick fever virus (strain USA/Florio N-7180)*	Q9DSQ0
	Cypovirus	*Bombyx cypovirus 1 (BMCV)*	AF323782.1
	Dinovernavirus	*Aedes pseudoscutellaris reovirus (isolate France)*	Q2Y0E9
	Fijivirus	*Fiji disease virus (Fijivirus)*	Q8JYK1
		Nilaparvata lugens reovirus (NFV)	NC_003654.1
	Mycoreovirus	*Cryphonectria parasitica mycoreovirus 1 (strain 9B21)*	Q7TDB6
	Orthoreovirus	*Reovirus type 1 (strain Lang)*	P0CK32
		Reovirus type 2 (strain D5/Jones)	P17377
		Reovirus type 3 (strain Dearing)	P0CK31
	Oryzavirus	*Rice ragged stunt virus (isolate Thailand)*	O92604
	Cardoreovirus	*Eriocheir sinensis reovirus (isolate China/905)*	Q698V5
	Mimoreovirus	*Micromonas pusilla reovirus (isolate Netherlands/2005)*	Q1I0V0
	Orbivirus	*Bluetongue virus 10*	P13840
	Phytoreovirus	*Rice gall dwarf virus (isolate Fujian)*	Q98631
		Rice dwarf virus (isolate Fujian)	Q98631
	Rotavirus	*Rotavirus A (isolate SI/South Africa/H96/58)*	A2T3S0
		Rotavirus B (isolate novel adult diarrhea rotavirus-J19)	Q45UG0
		Rotavirus C (isolate Human/United Kingdom/Bristol/1989)	Q91E95
	Seadornavirus	*Banna virus (strain Indonesia/JKT-6423/1980)*	Q9INJ1
Nodaviridae	*Betanodavirus*	*Striped jack nervous necrosis virus (STNV)*	Q9QAZ8
		Tiger puffer nervous necrosis virus (TPNV)	NC_013460
		Senegalese sole Iberian betanodavirus (SBIV)	NC_024492.1
	Alphanodavirus	*Flock house virus (FHV)*	Q66929
		Nodamura virus (NoV)	Q9IMM4
		Macrobrachium rosenbergii nodavirus (MrNV)	Q6XNL5
		Penaeus vannamei nodavirus (PVNV)	NC_014978.1
		Drosophila melanogaster American nodavirus (DmANV)	GQ342965.1
Unclassified	*Unasigned*	*Ixodes scapularis associated virus 2 (Ixodes2)*	KM048319.1
		Ixodes scapularis associated virus 1 (Ixodes1)	KM048318.1
Luteoviridae	*Polerovirus*	*Potato leafroll virus (PLRV)*	KC456054.1
		Cucurbit aphid-borne yellows vírus (CABYV)	NC_003688.1
	Enamovirus	*Pea enation mosaic virus-1 (strain WSG) (PEMV-1)*	P29154
		Citrus vein enation virus (CEMV)	YP_008130302.1
	Luteovirus	*Bean_leafroll_virus*	AAL66233.1
		Barley yellow dwarf virus (isolate PAV) (BYDV)	P09505
	Sobemovirus	*Soybean yellow common mosaic virus (SYCV)*	AEO16607
		Sesbania mosaic virus (SMV)	YP_007697678
	Tombusviridae	*Carnation mottle virus (CARMV)*	NC_001265.2
		Melon necrotic spot virus (MNSV)	AB232925.1
		Tobacco necrosis virus (TNV)	M33002.1

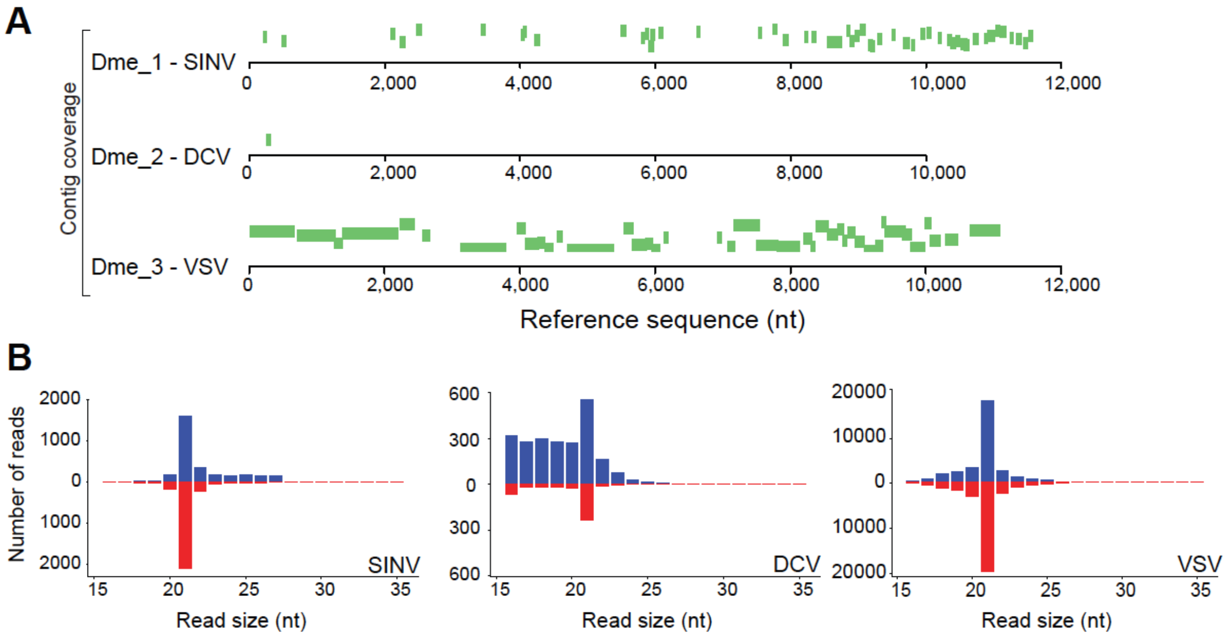

Supplementary Figure S1. Virus-derived small RNA can be assembled into long contig sequences. (**A**) Distribution of contigs assembled using our small RNA metagenomics strategy along the reference genomes of DCV, VSV and SINV. (**B**) Small RNA size profile of DCV, VSV and SINV utilized for laboratory infections in *Drosophila*.

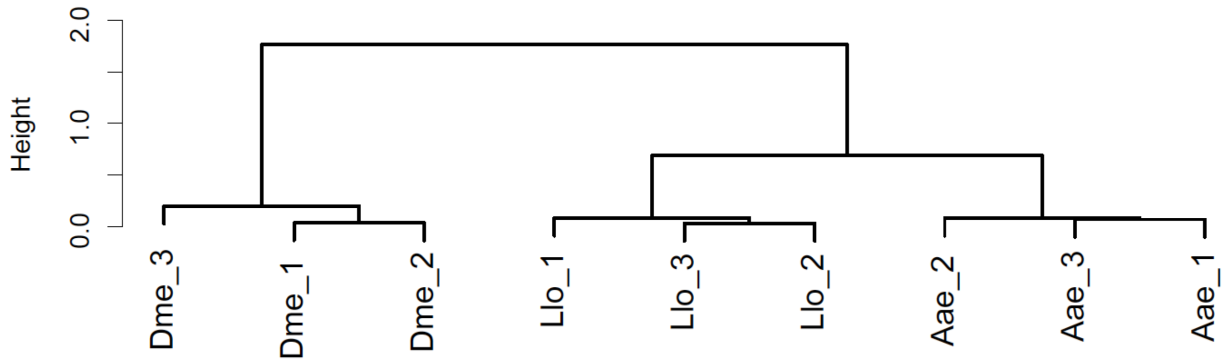

Supplementary Figure S2. Unknown contigs classify small RNA libraries in a host-specific manner. Clustering of libraries based on sequence similarity of unknown contigs is able to group libraries based on insect species. Contig clustering by similarity was performed using the BLASTClust program within the standalone BLAST package, requiring 50% of length with at least 50% of identity between contigs.

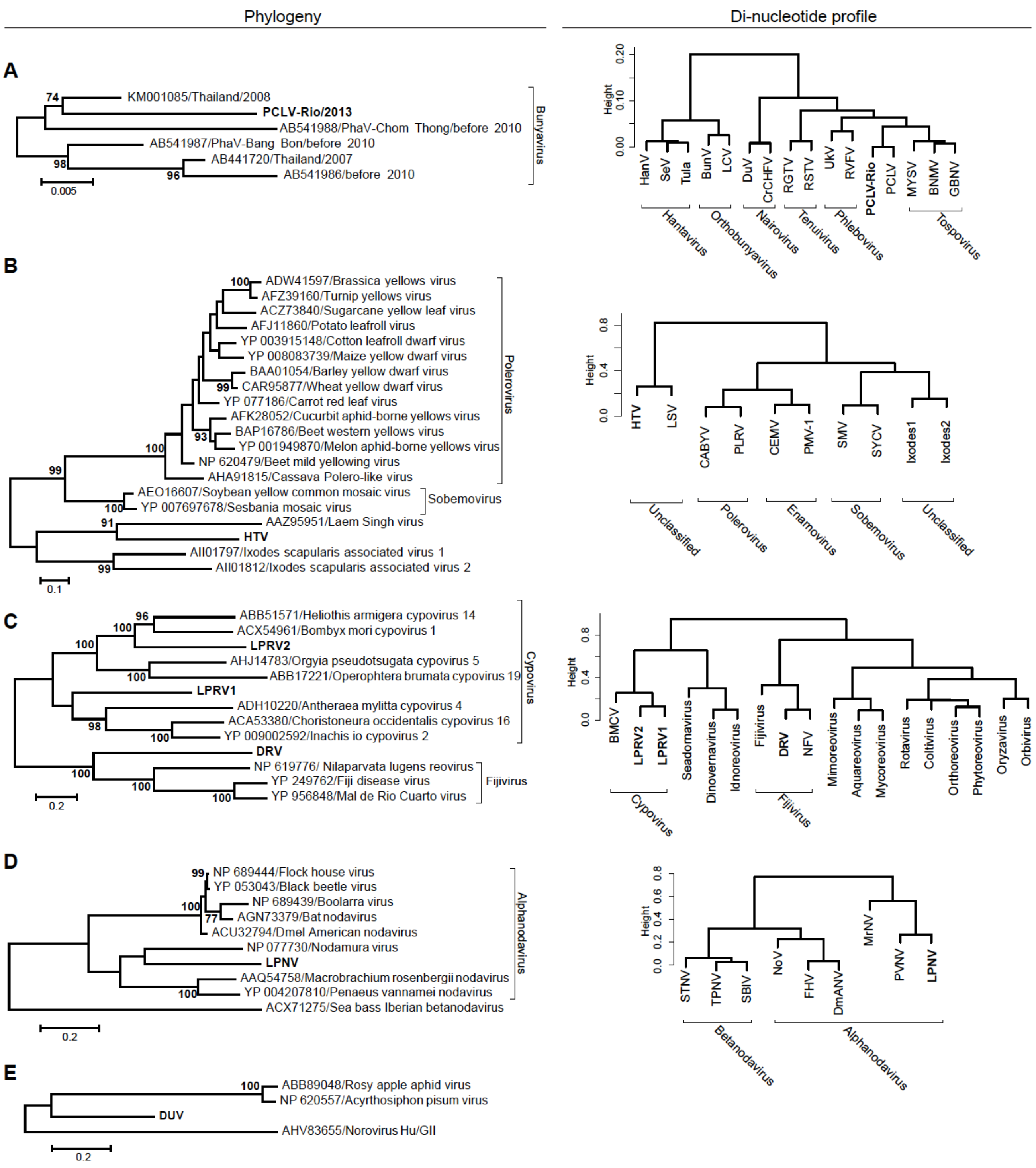

Supplementary Figure S3. Viruses identified by small RNA sequencing belong to diverse viral families. (**A**) PCLV in mosquitoes from Rio de Janeiro, Brazil clusters with other viral strains from Thailand on the phylogenetic tree with 74% bootstrap confidence at *p*-distance of 0.038. The dinucleotide profile reinforces the relationship between Rio and Asian strains of PCLV. (**B**) HTV also identified in mosquitoes

clusters with the unclassified *Laem Singh virus* (LSV) (also its top BLASTP hit) at 91% bootstrap confidence. The p-distance between HTV virus and *Laem Singh virus* is 0.57. The unclassified *Ixodes scapularis-associated viruses* 1 and 2 are the next nearest neighbours at p-distances of 0.68. The p-distances to the genera *Sobemovirus* and *Polerovirus* range from 0.72 to 0.77. Assignment of HTV virus to a genus is not possible, although we tentatively assign it to the *Luteoviridae* family. Dinucleotide analysis also clusters HTV and LSV together separate from other characterized viruses. (**C**) Two sandlfy reoviruses, LPRV1 and LPRV2, cluster within the genus *Cypovirus*, whereas the reovirus from fruit flies, DRV, appears as an outlier on the genus *Fijivirus*. We assign all three viruses to the family *Reoviridae*, sub-family *Spinareovirinae*. LPRV1 and LPRV2 have p-distances of 0.67 and 0.60 respectively to their nearest cypoviruses. The p-distance from DRV to the nearest Fijivirus is 0.75, and its p-distances to the cypoviruses range from 0.82 to 0.84. Dinucleotide analysis of reoviruses reinforces the classification suggested by phylogeny. (**D**) The sandfly virus, LPNV, clusters within the genus *Alphanodavirus* genus in the family *Nodaviridae*. The p-distance from LPNV to its top BLASTP hit, *Nodamura virus*, is 0.51, and its p-distances to the other members of genus *Alphanodavirus* range from 0.50 to 0.57. Its p-distance to the *Betanodavirus* outgroup sequence is 0.73. *p*-distances from the other members of genus *Alphanodaviruses* in the tree to the *Betanodavirus* outgroup are in the range 0.73 to 0.76. There is no bootstrap support for definition of a nearest neighbor for LPNV. Dinucleotide analysis reinforces the classification of LPNV within genus *Alphanodavirus*. (**E**) *Norovirus* is used as an outgroup as it has the third-best (but very poor) BLASTP hit to DUV found in fruit flies. The p-distance from DUV to both *Rosy apple aphid virus* and *Acyrthosiphon pisum virus* is 0.72. Both of these viruses are taxonomically unclassified and therefore no family assignment can be made for DUV.

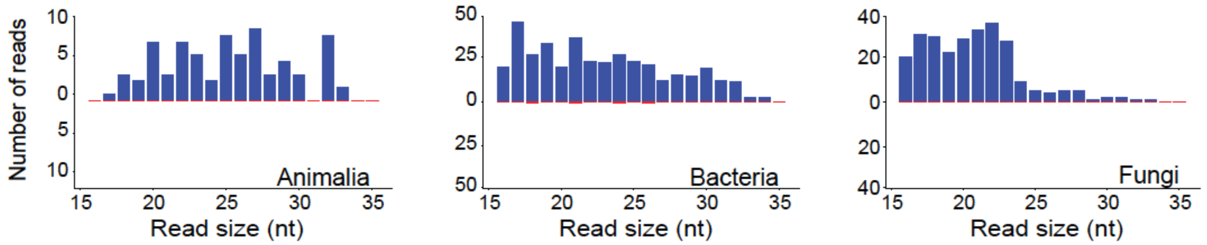

Supplementary Figure S4. Small RNA size profile of representative contigs derived from animal, bacterial and fungal sequences. The small RNA size profile of representative non-viral contigs derived from different taxa are shown. Blue and red represent small RNAs in positive and negative strands, respectively.

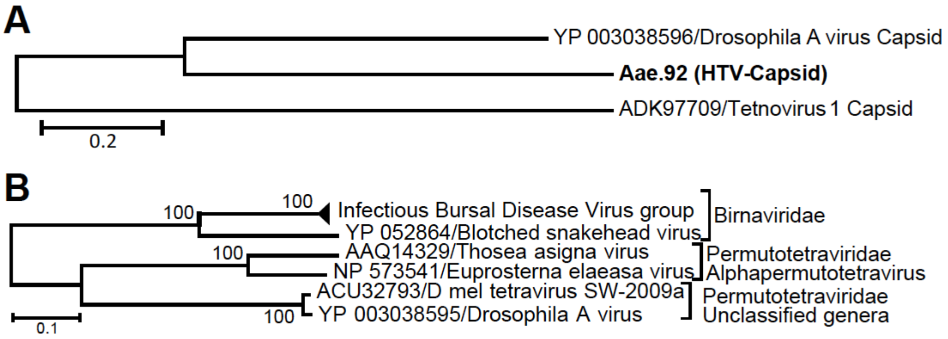

Supplementary Figure S5. Polymerase and capsid segments from HTV and DAV show similarity to different viral families. (**A**) Phylogenetic analysis of the HTV capsid and best BLASTP hits suggests it is more similar to DAV while HTV RdRP is most similar to other uncharacterized viruses and the *Luteoviridae* family (**Supplementary Figure S3B**). *Tetnovirus 1* is used as an outgroup as it has the third-best (but very poor) BLASTP hit to HTV capsid. (**B**) Phylogenetic analysis of the DAV RdRP and best BLASTP hits suggests it is more similar to viruses from the family *Permutotetraviridae,* 100% bootstrap at *p*-distance of 0.0009 to *Drosophila melanogaster tetravirus,*.

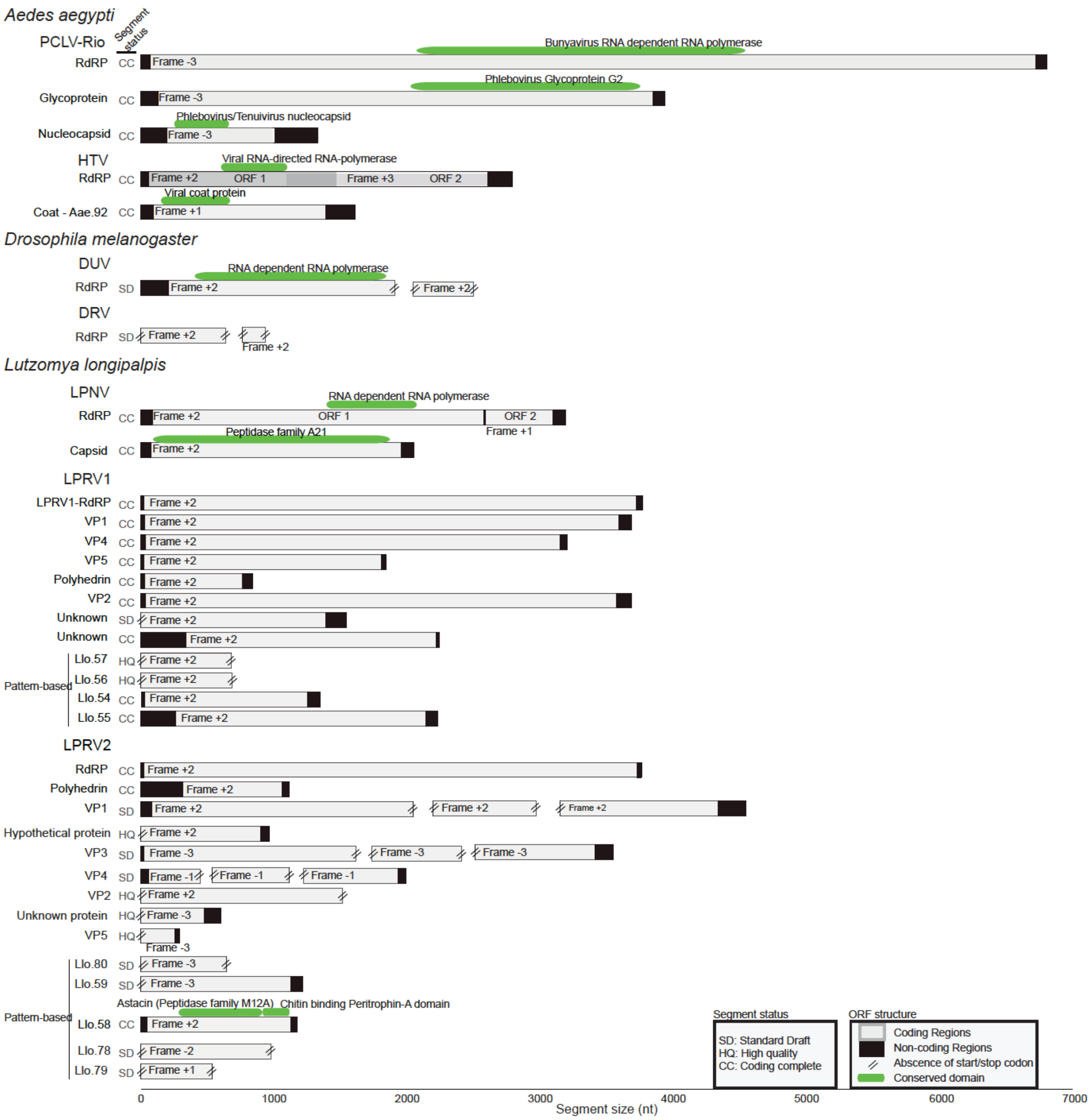

Supplementary Figure S6. ORF organization and domain analysis of viral sequences identified by our strategy. Viral contigs are shown in the same scale highlighting predicted ORFs and conserved protein domains. The status of the virus sequence relative to the expected complete genome and presence of start/stop codons are shown. Annotation status of novel viral sequences followed the guidelines previously described by Ladner et al (39).

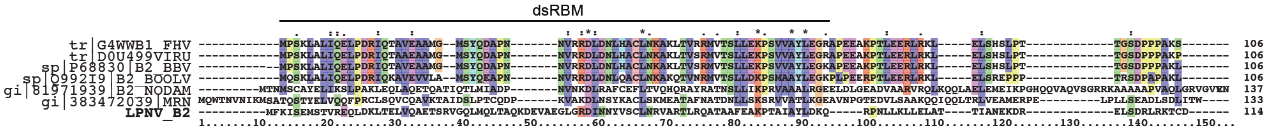

Supplementary Figure S7. Analysis of B2-like protein from LPNV. Protein sequence alignment between a putative protein encoded by RNA 1 of LPNV and other B2 proteins derived from Alphanodaviruses. Despite high conservation, protein alignment of suggests the LPNV B2-like protein lacks several conserved residues within the putative dsRNA Binding Motif (dsRBM)**.**

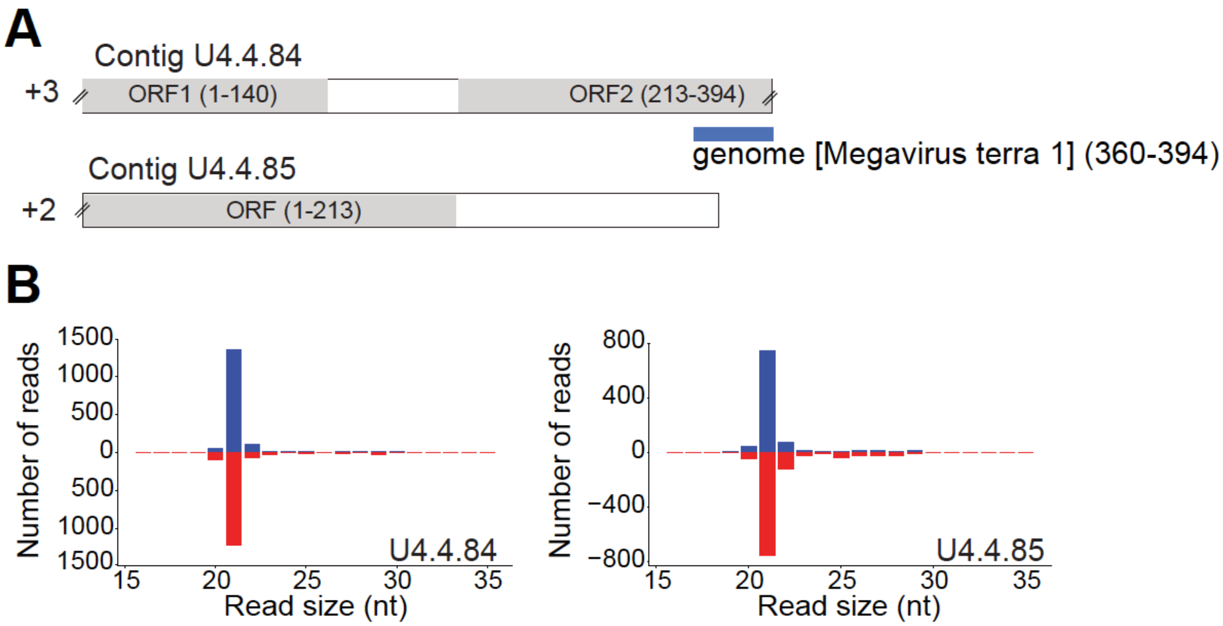

Supplementary Figure S8. ORF structure and small RNA profile of unknown viral sequences identified in small RNA libraries from U4.4 cells. (**A**) Contigs U4.4.84 and U4.4.85 have incomplete ORFs with no conserved domains. The second incomplete ORF of contig U4.4.84 shows similarity to *Megavirus terra 1*. (**B**) Small RNAs with a canonical siRNA profile mapping to contigs U4.4.84 and U4.4.85 are also found in libraries from Aag2 cells prepared in the same laboratory.

SUPPLEMENTARY REFERENCES

62. Edgar, R.C. (2004) MUSCLE: multiple sequence alignment with high accuracy and high throughput. *Nucleic acids research*, **32**, 1792-1797.

63. Tamura, K., Stecher, G., Peterson, D., Filipski, A. and Kumar, S. (2013) MEGA6: Molecular Evolutionary Genetics Analysis version 6.0. *Molecular biology and evolution*, **30**, 2725-2729.

64. Tamura, K. (1992) Estimation of the number of nucleotide substitutions when there are strong transition-transversion and G+C-content biases. *Molecular biology and evolution*, **9**, 678-687.

65. Whelan, S. and Goldman, N. (2001) A general empirical model of protein evolution derived from multiple protein families using a maximum-likelihood approach. *Molecular biology and evolution*, **18**, 691-699.

66. Zuckerkandl, E. and Pauling, L. (1965) Molecules as documents of evolutionary history. *Journal of theoretical biology*, **8**, 357-366.

67. Lobo, F.P., Mota, B.E., Pena, S.D., Azevedo, V., Macedo, A.M., Tauch, A., Machado, C.R. and Franco, G.R. (2009) Virus-host coevolution: common patterns of nucleotide motif usage in Flaviviridae and their hosts. *PloS one*, **4**, e6282.
